# Water coordinated on Cu(I)-based catalysts is the oxygen source in CO_2_ reduction to CO

**DOI:** 10.1038/s41467-022-30289-5

**Published:** 2022-05-11

**Authors:** Yajun Zheng, Hedan Yao, Ruinan Di, Zhicheng Xiang, Qiang Wang, Fangfang Lu, Yu Li, Guangxing Yang, Qiang Ma, Zhiping Zhang

**Affiliations:** 1 School of Chemistry and Chemical Engineering, Xi’an Shiyou University, Xi’an, 710065 China; 2 School of Chemistry and Molecular Engineering, Nanjing Tech University, Nanjing, 211816 China; 3 School of Chemistry and Chemical Engineering, South China University of Technology, Guangzhou, 510641 China; 4 Chinese Academy of Inspection and Quarantine, Beijing, 100176 China

**Keywords:** Mass spectrometry, Electrocatalysis, Electrocatalysis

## Abstract

Catalytic reduction of CO_2_ over Cu-based catalysts can produce various carbon-based products such as the critical intermediate CO, yet significant challenges remain in shedding light on the underlying mechanisms. Here, we develop a modified triple-stage quadrupole mass spectrometer to monitor the reduction of CO_2_ to CO in the gas phase online. Our experimental observations reveal that the coordinated H_2_O on Cu(I)-based catalysts promotes CO_2_ adsorption and reduction to CO, and the resulting efficiencies are two orders of magnitude higher than those without H_2_O. Isotope-labeling studies render compelling evidence that the O atom in produced CO originates from the coordinated H_2_O on catalysts, rather than CO_2_ itself. Combining experimental observations and computational calculations with density functional theory, we propose a detailed reaction mechanism of CO_2_ reduction to CO over Cu(I)-based catalysts with coordinated H_2_O. This study offers an effective method to reveal the vital roles of H_2_O in promoting metal catalysts to CO_2_ reduction.

## Abstract (web summary)

Understanding the underlying mechanisms for catalytic reduction of CO_2_ over Cu based catalysts remains challenging. Here, the authors develop an effective method to reveal the vital roles of H_2_O in promoting metal catalysts to CO_2_ reduction via a modified triple stage quadrupole mass spectrometer.

## Introduction

Catalytic reduction of CO_2_ into high value-added carbon-based products is a promising strategy for tackling current energy demands and reducing greenhouse gas emissions^1–5^. In the past few years, tremendous efforts have been made to explore CO_2_ reduction reaction (CO_2_RR), and several products, including CH_4_^6^, CO^7^, CH_3_OH^8^, HCOOH^9^, HCHO^10^, C_2_H_4_^11^, C_2_H_6_^12^, C_2_H_5_OH^13^, and H_2_C_2_O_4_^14^, have been generated from CO_2_ reduction via photo-, electro-, or thermal activation^3–5,15–18^. To enhance the selectivity and the conversion efficiency of CO_2_RR, focus has primarily been on exploring novel catalysts^2,3,19,20^. Despite the progress, many details of the CO_2_RR mechanisms on the surface of catalysts remain elusive. The techniques used to investigate CO_2_RR mechanisms include Raman spectroscopy^21^, X-ray absorption spectroscopy^22^, X-ray photoelectron spectroscopy^23^, electron microscopy^24^, and calculations using density functional theory (DFT)^25,26^. However, direct observation of the highly reactive intermediates is still a grand challenge.

**Fig. 1:**
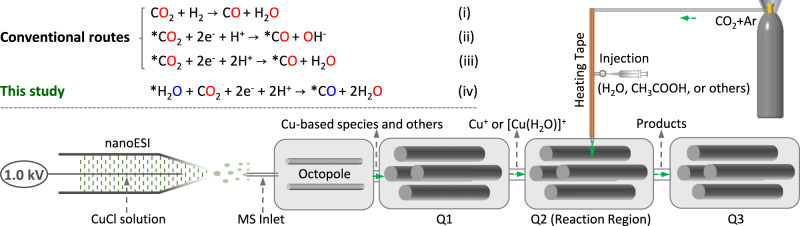
Schematic diagram of the apparatus for CO_2_ reduction and detection of reaction products. (insets in the top left corner are the different routes for generation of CO from CO_2_, in which * means catalyst).

Mass spectrometry (MS) is a formidable tool for chemical analysis and has also been used to explore the mechanisms of various chemical reactions^27–31^. Nevertheless, there have been few reports on the use of MS to study CO_2_RR mechanisms in operando^32^. This could be attributed to the following obstacles: (i) CO_2_ and its resulting products (e.g., CO, CH_4_, and CH_3_OH) are neutral molecules and rarely observed in mass spectra without adding charges through ionization; (ii) the lifetimes of reactive intermediates are typically less than milliseconds, making it challenging to capture them with off-line MS. To address these issues, this study proposes a strategy to explore CO_2_RR mechanisms by adopting the features of triple-stage quadrupole (TSQ) mass spectrometer, in which different stages of quadrupoles in TSQ are used for separation of metal ions and related catalysts originating from nanoelectrospray ionization (nanoESI)^33^ (first stage), reaction unit of metal catalysts and CO_2_ (second stage), and transmission of resulting ions (third stage) (Fig. 1). In the second stage, CO_2_ and its resulting products could be charged favorably by interacting with metal ions and forming metal complexes. More importantly, the CO_2_RR processes can be simultaneously carried out with online detection. As such, the reaction pathways of CO_2_RR can be well identified.

To demonstrate the proof-of-concept, this study explores the reduction reaction of CO_2_ to CO using copper (Cu) as the catalyst. Cu is the only metal catalyst known to generate hydrocarbons through CO_2_RR^5,34–39^, but poor selectivity and reaction efficiency for value-added products significantly limit its commercial applications^6,40^. Although much effort has been made to understand the catalytic performance of Cu in CO_2_RR, available techniques that can clarify the underlying reaction mechanisms remain limited^41^. In the CO_2_RR, CO has been widely identified as a significant product or a primary reaction intermediate for the formation of hydrocarbons^15–17,42,43^. Therefore, understanding the reduction reaction of CO_2_ to CO over Cu-based catalysts is necessary to promote conversion efficiency.

According to general understanding, there are three routes in CO_2_ reduction to CO: (i) CO_2_ + H_2_ → CO + H_2_O^1,17,44^, (ii) *CO_2_ + 2e^−^ + H^+^ → CO + OH^−^^45^, and (iii) *CO_2_ + 2e^−^ + 2H^+^ → CO + H_2_O^43,46–50^ (Fig. 1). For all three pathways, the O atom in the generated CO originated from CO_2_. In contrast to the above routes, herein we discovered for the first time that the source of the O atom in CO originated from the H_2_O coordinated on transition metal-based catalysts (e.g., Cu, Ag, and Pd) rather than CO_2_. H_2_O is ubiquitous in nature and has been shown to be vital in many reactions such as alcohol oxidation to aldehyde^51^, CH_4_ oxidation to CH_3_OH^52,53^, and CO_2_ reduction to CH_4_, CH_3_OH, and HCOOH^54^. However, the role of H_2_O in the reduction of CO_2_ to CO is ambiguous. To elucidate the role of H_2_O in CO_2_RR, we investigated the behavior of Cu^+^ and [Cu(H_2_O)]^+^ and found that the coordinated H_2_O in [Cu(H_2_O)]^+^ not only favored the adsorption of CO_2_ onto Cu^+^, but also facilitated the reduction of CO_2_ to CO. Isotope-labeling studies provided evidence suggesting that the origin of the O atom in CO was from the coordinated H_2_O on Cu^+^. By combining experimental results and computational calculations with DFT, the detailed reaction mechanism of CO_2_ reduction to CO over a [Cu(H_2_O)]^+^ catalyst was proposed. The data presented in this work allowed us to elucidate the role of H_2_O in CO_2_RR and offered new insights for developing effective systems that enhanced the selectivity and conversion efficiency of CO_2_ to CO and other carbon-based products.

## Results and Discussion

### Reaction apparatus and generation of Cu(I) species

To reveal the underlying roles of H_2_O in the reduction of CO_2_ to CO via copper-based catalysis, we employed a modified TSQ apparatus for online observation of the CO_2_RR and detection of the reaction intermediates and products. As shown in Fig. 1, nanoESI was used as an ionization source to generate Cu-based ions. As the catalyst ions were introduced into the TSQ apparatus, the desired Cu species were isolated from Q1 and then transferred to Q2. In the reaction unit of Q2, Cu-based ions interacted with CO_2_ upon the applied voltage of 5 V and formed CO. Due to the low reaction efficiency at low temperatures (as discussed below), CO_2_ gas was heated to 280 °C using a heating tape in the gas circuit system prior to reacting with the Cu catalyst in the gas phase. After the reduction reaction was completed, the resulting products were transferred directly to Q3 followed by online detection.

Many previous studies^8,26,55–59^ have demonstrated that of the different oxidation states, Cu(I)-related species are one of the most important catalysts in CO_2_RR. Considering that the current study investigated different Cu-based complexes in the reduction of CO_2_, nanoESI was utilized to generate Cu(I) species rather than inductively coupled plasma (ICP)^60,61^, which only produces Cu(I). In the nanoESI process, the types of Cu species and solvent were found to have a pronounced effect on the resulting mass spectra. When CuX (X = Cl, Br, or I) was dissolved into acetonitrile, comparable and intensive peaks of Cu-based ions appeared in the mass spectra (e.g., ^63^Cu^+^, ^65^Cu^+^, [^63^Cu(H_2_O)]^+^, and [^65^Cu(H_2_O)]^+^), and few non-Cu species emerged (Supplementary Figs. 1–3). Thus, CuCl was used to generate different Cu(I)-based ions.

### Effect of H_2_O on the reduction of CO_2_ to CO

**Fig. 2:**
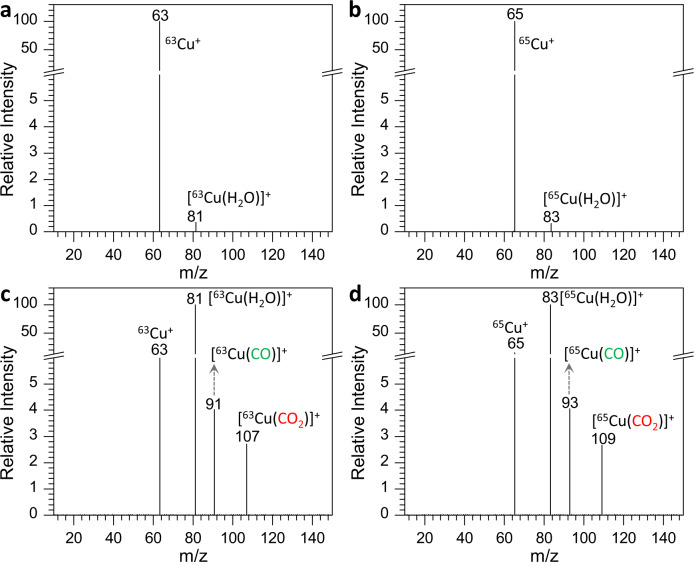
Mass spectra of CO_2_ reduction to CO under different Cu-based catalytic systems. **a** ^63^Cu^+^ **b** ^65^Cu^+^. **c** [^63^Cu(H_2_O)]^+^. **d** [^65^Cu(H_2_O)]^+^ (gas circuit temperature: 280 ^o^C; reaction pressure: 1.5 mTorr).

The above MS setup allowed us to investigate the reduction of CO_2_ to CO in situ. As ^63^Cu^+^ or ^65^Cu^+^ ions were isolated to interact with CO_2_, no obvious resultant species were observed, and only Cu^+^ itself and [Cu(H_2_O)]^+^ appeared in the mass spectra (Fig. 2a, b). Remarkably, when [^63^Cu(H_2_O)]^+^ or [^65^Cu(H_2_O)]^+^ was introduced to the reaction unit along with CO_2_, not only did the reaction product, CO, emerge in the forms of [^63^Cu(CO)]^+^ and [^65^Cu(CO)]^+^, but so did the reactant, CO_2_, as complexes of [^63^Cu(CO_2_)]^+^ and [^65^Cu(CO_2_)]^+^, in addition to [^63^Cu(H_2_O)]^+^, [^65^Cu(H_2_O)]^+^, and their fragment ions ^63^Cu^+^ and ^65^Cu^+^ (Fig. 2c, d). These results suggested that in contrast to the bare Cu^+^, the coordinated H_2_O in [Cu(H_2_O)]^+^ played a vital role in the interaction with CO_2_, which both rendered the adsorption of CO_2_ and the reduction of CO_2_ to CO on the Cu(I) catalyst. We also found that the coordinated H_2_O favored other metal-based ions (e.g., [Ag(H_2_O)]^+^ and [Pd(H_2_O)]^+^) to carry out the CO_2_RR (Supplementary Figs. 4 and 5), revealing that H_2_O molecules on the metal catalysts were active sites for facilitating the effective reduction of CO_2_ to CO. The effect of H_2_O could also be mirrored by a reversed water-gas shift reaction (WGSR) using Cu/ZnO/Al_2_O_3_ as catalyst (Supplementary Fig. 6a–c) and in situ diffuse reflectance infrared Fourier transform spectroscopy (in situ DRIFTS) using Cu/γ-Al_2_O_3_ and Pt/γ-Al_2_O_3_ as catalysts (Supplementary Fig. 7). It hinted that dosing a suitable amount of H_2_O could promote heterogeneous thermal catalytic conversion of CO_2_ to CO in realistic conditions.

**Fig. 3:**
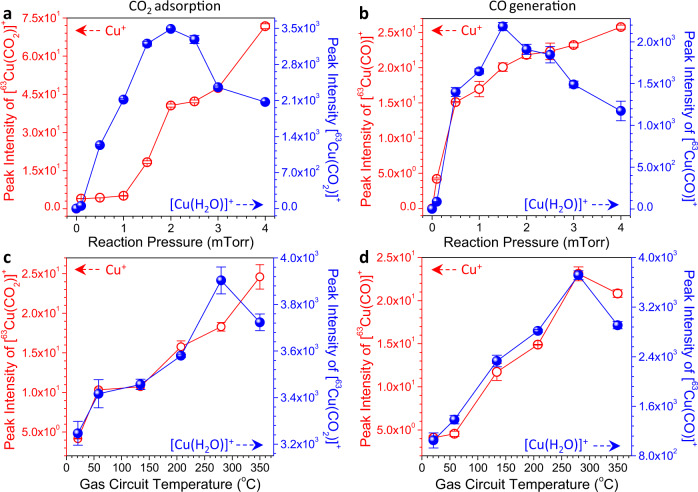
Variation of CO_2_ adsorption and CO generation with reaction pressure and temperature of heating tape around the gas circuit. Effect of reaction pressure on (**a**) the adsorption of CO_2_ onto ^63^Cu^+^ and (**b**) CO generation by CO_2_ reduction in the presence of either ^63^Cu^+^ or [^63^Cu(H_2_O)]^+^ catalysts (gas circuit temperature: 280 ^o^C). Effect of the gas circuit temperature on (**c**) the adsorption of CO_2_ onto Cu^+^ and (**d**) CO generation by CO_2_ reduction in the presence of either ^63^Cu^+^ or [^63^Cu(H_2_O)]^+^ catalysts (reaction pressure: 1.5 mTorr).

To quantitatively describe the roles of coordinated H_2_O over metal surfaces to CO_2_ adsorption and reduction to CO, we used Cu-based catalysts as an example. Fig. 3a and Supplementary Fig. 8a show the effect of reaction gas pressure on the adsorption of CO_2_ to Cu^+^. The adsorption performance was examined by the absolute peak intensities of generated [Cu(CO_2_)]^+^ in mass spectrometric analysis. When bare Cu^+^ was employed, the adsorption ability of CO_2_ onto the Cu^+^ catalyst gradually increased with the increasing reaction gas pressure from 0 to 4 mTorr. However, in the presence of [Cu(H_2_O)]^+^, the adsorption of CO_2_ onto Cu^+^ increased with increasing reaction gas pressure and reached a maximum value in the range of 1.5 – 2.5 mTorr, then decreased thereafter. More interestingly, in the presence of [^63^Cu(H_2_O)]^+^, the peak intensity of [^63^Cu(CO_2_)]^+^ was 48.6-fold higher than that with ^63^Cu^+^ in their optimal conditions. This demonstrated that after H_2_O was coordinated to Cu^+^, CO_2_ was more prone to interact with [^63^Cu(H_2_O)]^+^ to generate [^63^Cu(CO_2_)]^+^ compared to bare Cu^+^. Besides CO_2_, we also found that in contrast to Cu^+^, [Cu(H_2_O)]^+^ was likely to form Cu-based complexes with other molecules such as methanol (Supplementary Fig. 9), ethanol (Supplementary Fig. 10), acetonitrile (Supplementary Fig. 11), benzene (Supplementary Fig. 12), toluene (Supplementary Fig. 13), and dichloromethane (Supplementary Fig. 14). Such a fact suggests that the coordinated H_2_O in the structure of [Cu(H_2_O)]^+^ is a uniquely active site for adsorbing different molecules. To our knowledge, it could be speculated as the following reasons. As H_2_O was bound to Cu^+^, the resulting [Cu(H_2_O)]^+^ more likely tended to form hydrogen bond^62–66^ or OH-π interactions^67–69^ with those studied molecules than bare Cu^+^. After undergoing further structural rearrangements, Cu(I)-based complexes were favorably generated. Despite this, detailed reasons need to be further studied.

It is well known that adsorption onto catalyst surfaces is a very crucial step for CO_2_ reduction, and a more favorable CO_2_ adsorption can facilitate a more efficient reduction to CO^15^. To evaluate the reduction ability of CO_2_ to CO in the presence of Cu^+^ and [Cu(H_2_O)]^+^, we compared the difference in quantity of generated [Cu(CO)]^+^ at varying gas pressures (Fig. 3b and Supplementary Fig. 8b). For bare Cu^+^, the peak intensity of [^63^Cu(CO)]^+^ sharply increased at 0 – 0.5 mTorr, followed by a gradual increase at 1 – 4 mTorr. A different pattern was observed for [Cu(H_2_O)]^+^, where the peak intensity first increased to a maximum value at 1.5 mTorr, and then decreased thereafter. Comparing both systems with Cu^+^ and [Cu(H_2_O)]^+^, it was remarkable that [Cu(CO)]^+^ was generated more favorably for [Cu(H_2_O)]^+^. Similar to the amount of adsorbed CO_2_, the amount of CO generated in the presence of [^63^Cu(H_2_O)]^+^ was 84.7-fold higher than that in the presence of ^63^Cu^+^ under optimal conditions. These results indicated that the coordinated H_2_O in [^63^Cu(H_2_O)]^+^ not only greatly facilitated CO_2_ adsorption to Cu^+^, but also significantly promoted CO generation.

A similar process was also conducted to compare the adsorption of CO_2_ and generation of CO at different temperatures, which was controlled by varying the temperature of heating tape around the gas circuit (Fig. 1). As the temperature increased from room temperature (20 °C) to 350 °C, CO_2_ adsorption using ^63^Cu^+^ demonstrated a gradual increasing pattern. However, a different pattern was observed for the system using [^63^Cu(H_2_O)]^+^, in which an initial steady increasing trend from room temperature to 280 °C was observed, followed by a slight decreasing one within the range of 280 – 350 °C (Fig. 3c and Supplementary Fig. 8c). In the optimal temperature range, the CO_2_ adsorption amount in the presence of [^63^Cu(H_2_O)]^+^ was more than two orders of magnitude (158.7-fold) higher than ^63^Cu^+^. For the generation of CO, both ^63^Cu^+^ and [^63^Cu(H_2_O)]^+^ catalytic systems demonstrated a comparable pattern, in which the formation of CO first increased and reached a maximum value at 280 °C, then decreased thereafter (Fig. 3d and Supplementary Fig. 8d). By comparing both systems, it was found that the use of [^63^Cu(H_2_O)]^+^ resulted in more than two orders of magnitude (160.9-fold) of CO formation than using ^63^Cu^+^. Thus, we can conclude in confident that the coordinated H_2_O in [^63^Cu(H_2_O)]^+^ was a governing factor in CO_2_ adsorption and CO generation.

From the above, it was noticeable that there was a maximum CO_2_ adsorption capacity and a maximum CO production rate when [Cu(H_2_O)]^+^ was used as catalyst throughout the studied temperature and pressure ranges (Fig. 3). This phenomenon was presumably due to the instability of [Cu(H_2_O)]^+^ with increasing reaction pressures and temperatures. As an example of increasing reaction pressures, the content of CO_2_ in the Q2 of mass spectrometer (Fig. 1) steadily increased and therefore, high yields of [Cu(CO_2_)]^+^ and [Cu(CO)]^+^ should be generated, whereas an opposite trend was observed within the range of 2 – 4 mTorr (Fig. 3a, b). As aforementioned, [Cu(H_2_O)]^+^ was more favorable for the generation of [Cu(CO_2_)]^+^ and [Cu(CO)]^+^ than Cu^+^. However, the generated amount of [Cu(H_2_O)]^+^ demonstrated a decreasing pattern with increasing reaction pressures from 2 to 4 mTorr (Supplementary Fig. 15a). A lower amount of [Cu(H_2_O)]^+^ would result in a lower generation efficiency to [Cu(CO_2_)]^+^ and [Cu(CO)]^+^. As a compromise between reaction pressure and the amount of [Cu(H_2_O)]^+^, 1.5 mTorr gave the optimal performance. As to the temperature, the same pattern as the reaction pressure was observed (Fig. 3c, d), and 280 °C offered the highest generation efficiencies to both [Cu(CO_2_)]^+^ and [Cu(CO)]^+^. This case may be associated with the compromise of [Cu(H_2_O)]^+^ stability (Supplementary Fig. 15b) and the thermodynamic reaction activity between CO_2_ and [Cu(H_2_O)]^+^ under increasing temperatures.

### Origin of the O atom in generated CO

Isotope-labeling experiments (H_2_^16^O, H_2_^18^O, C^16^O_2_, and C^18^O_2_) allowed us to conclude that the origin of the O atom in the generated CO was from the coordinated H_2_O, rather than CO_2_ itself. In previous studies^1,17,34,43–50^, different mechanistic scenarios were proposed to elucidate the reduction pathway of CO_2_ to CO, and the generation of CO was conventionally contributed to the loss of an O atom in CO_2_. However, no direct experimental evidence generated through isotopic labeling has been supported thus so far. MS has been demonstrated to be a very powerful tool that offers the opportunity to derive the origin of the O atom in generated CO from CO_2_.

**Fig. 4:**
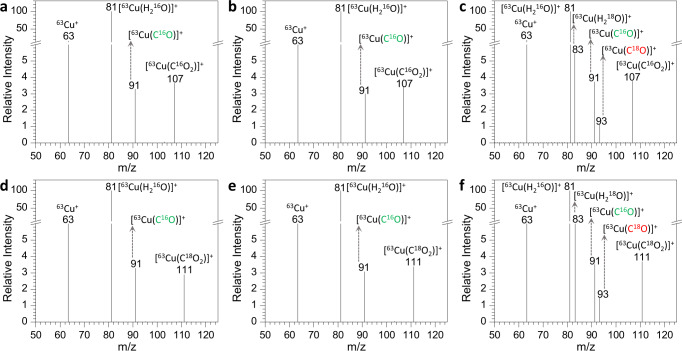
Isotope-labeling MS measurement results under different systems. **a** [^63^Cu(H_2_^16^O)]^+^ and C^16^O_2_. **b** [^63^Cu(H_2_^16^O)]^+^, H_2_^16^O, and C^16^O_2_. **c** [^63^Cu(H_2_^16^O)]^+^, H_2_^18^O, and C^16^O_2_. **d** [^63^Cu(H_2_^16^O)]^+^ and C^18^O_2_. **e** [^63^Cu(H_2_^16^O)]^+^. H_2_^16^O, and C^18^O_2_; **f** [^63^Cu(H_2_^16^O)]^+^, H_2_^18^O and C^18^O_2_ (gas circuit temperature: 280 ^o^C; reaction pressure: 1.5 mTorr).

As expected, monitoring of the reaction between [^63^Cu(H_2_^16^O)]^+^ and C^16^O_2_ resulted in the observation of [^63^Cu(C^16^O)]^+^ (Fig. 4a). The inability to isolate [^63^Cu(H_2_^18^O)]^+^, by dissolving CuCl or other Cu-based chemicals into the mixture of acetonitrile and H_2_^18^O, meant that it was impossible to probe the reaction products in the presence of [^63^Cu(H_2_^18^O)]^+^ and C^16^O_2_. This case could be attributable to the fact that there are plenty of H_2_^16^O in air, and the H_2_^18^O in generated [^63^Cu(H_2_^18^O)]^+^ would be quickly exchanged by H_2_^16^O in the plume of nanoESI or the transfer process to the Q1 of mass spectrometer (Fig. 1). As a result, insufficient [^63^Cu(H_2_^18^O)]^+^ ions could be generated and were not favorable for MS detection. To resolve this issue, [^63^Cu(H_2_^16^O)]^+^ was first isolated in Q1, and H_2_^16^O or H_2_^18^O was then injected into the CO_2_ gas circuit (Fig. 1). After interaction among the reactants in the reaction region Q2, the resulting products were analyzed. As H_2_^16^O was injected (Fig. 4b), the mass spectrum was analogous to that of only [^63^Cu(H_2_^16^O)]^+^ and C^16^O_2_ (Fig. 4a). Noticeably, when H_2_^18^O was added to the system containing [^63^Cu(H_2_^16^O)]^+^ and C^16^O_2_ (Fig. 4c), not only did [^63^Cu(H_2_^18^O)]^+^ appear in the mass spectrum, but so did [^63^Cu(C^16^O)]^+^ and [^63^Cu(C^18^O)]^+^. These results indicated that it was an effective strategy for generating [^63^Cu(H_2_^18^O)]^+^, and more importantly, the origin of the O atom in CO was presumable from the coordinated water.

More convincing evidence was gained from the reaction between [^63^Cu(H_2_^16^O)]^+^, C^18^O_2_, and H_2_^16^O/H_2_^18^O. Interestingly, as [^63^Cu(H_2_^16^O)]^+^ interacted with C^18^O_2_ (Fig. 4d), [^63^Cu(C^16^O)]^+^, rather than [^63^Cu(C^18^O)]^+^, was formed, indicating that the O atom in CO was indeed from the H_2_O. The same result was obtained from the system with [^63^Cu(H_2_^16^O)]^+^, C^18^O_2_, and H_2_^16^O (Fig. 4e). However, as H_2_^18^O was injected into the C^18^O_2_ gas circuit system (Fig. 4f), the mass spectrum was analogous to the system with [^63^Cu(H_2_^16^O)]^+^, C^16^O_2_ and H_2_^18^O (Fig. 4c), in which both [^63^Cu(C^16^O)]^+^ and [^63^Cu(C^18^O)]^+^ were generated. This phenomenon, as well as ^65^Cu (Supplementary Fig. 16), ^107^Ag, and ^104^Pd (Supplementary Fig. 17)-based reactions, allowed us to draw a definite conclusion that in the CO_2_RR process, the two O atoms in CO_2_ were eliminated completely by losses of two molecules of H_2_O^46,48,70^, and the O atom in CO originated from the coordinated H_2_O. Moreover, H_2_^18^O-labeling experiments in tandem with off-line GC-MS analysis were carried out to confirm the source of O atom in resulting CO from the reversed WGSR. As shown in Supplementary Fig. 6f–h, in comparison with the system of introducing H_2_^16^O, an abundant peak of m/z 30 (C^18^O) emerged in the mass spectrum for the system of H_2_^18^O. This fact indicated that the involved H_2_O was the oxygen source of the resulting CO. More importantly, the observed results of CO_2_ reduction in the TSQ mass spectrometer could be extrapolated to realistic heterogeneous catalysis. Those gas-phase results also correlated directly to solution-phase CO_2_RR. To gain insight into this point, we performed the electrochemical reduction of CO_2_ in KCl aqueous solution using Au electrode, because the produced amount of CO was below the limit of detection using Cu or Ag electrode. The reaction products were monitored using an online differential electrochemical mass spectrometer (Supplementary Fig. 18). In contrast to the system of H_2_^16^O, a considerable amount of C^18^O (m/z 30) was generated in the presence of H_2_^18^O (Supplementary Figs. 19–21). This further indicated that H_2_O was the oxygen source of resulting CO from CO_2_ reduction and provided solid evidence on the generalization of the current finding to related reactions in the condensed phase (Supplementary Table 1).

### Identifying the effect of H_2_O on CO generation

**Fig. 5:**
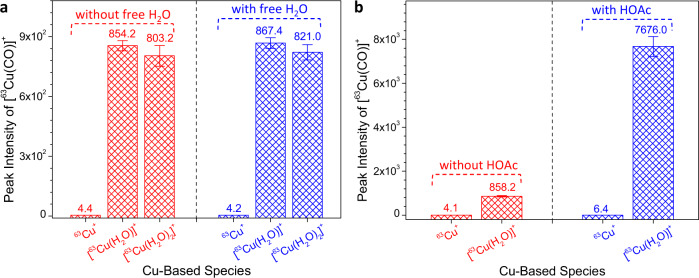
Influence of the number of coordinated H_2_O, free H_2_O, and extraneous acid on the generation of CO. **a** Effects of the number of coordinated H_2_O and free H_2_O on CO_2_ reduction to CO under different Cu-based catalytic systems (^63^Cu^+^, [^63^Cu(H_2_O)]^+^, and [^63^Cu(H_2_O)_2_]^+^). **b** Effect of extraneous acid on the reduction of CO_2_ to CO under different Cu-based catalytic systems. Note: The free H_2_O and HOAc (acetic acid) were injected into the gas circuit system by an injector; gas circuit temperature: 280 ^o^C; reaction pressure: 1.5 mTorr; *n* = 5).

The number of coordinated H_2_O in [^63^Cu(H_2_O)_x_]^+^ (*x* = 0, 1, or 2) was observed to have a significant influence on the reduction of CO_2_ to CO. The reaction efficiency was evaluated by comparing the absolute peak intensities of generated [^63^Cu(CO)]^+^ in mass spectrometric analysis. When bare ^63^Cu^+^ was used as the catalyst, a low signal was observed (Fig. 5a and Supplementary Fig. 22). Noticeably, a significant improvement was observed for [^63^Cu(H_2_O)]^+^, and the reaction efficiency was 193.8 times higher than with ^63^Cu^+^. However, further increasing the number of coordinated H_2_O to 2 (e.g., [^63^Cu(H_2_O)_2_]^+^) resulted in a comparable reaction efficiency to [^63^Cu(H_2_O)]^+^, indicating that one coordinated H_2_O molecule was sufficient for high-efficiency reduction of CO_2_ to CO.

In the reaction between [^63^Cu(H_2_O)]^+^ and CO_2_, it was unclear how H_2_O molecules interacted with CO_2_, namely whether it was a (i) direct interaction between [^63^Cu(H_2_O)]^+^ and CO_2_, or if (ii) CO_2_ first interacted with the dissociated H_2_O molecule from [^63^Cu(H_2_O)]^+^, then reacted with dissociated ^63^Cu^+^ or [^63^Cu(H_2_O)]^+^. To probe the above assumptions, 2 μL of free H_2_O was injected into the CO_2_ gas circuit system at a temperature of 280 °C (Fig. 1). After being injected, the free H_2_O molecules immediately evaporated and interacted with CO_2_ in the gas circuit by forming H_2_CO_3_ (H_2_O + CO_2_ → H_2_CO_3_)^71–73^, which was indirectly confirmed by the isotope-labeling experiments, namely H_2_^18^O and C^16^O_2_ (Supplementary Fig. 23). If route (ii) was taken in the CO_2_RR, the involved free H_2_O would facilitate CO_2_ reduction to CO. However, a comparable reaction efficiency to the system without free H_2_O was observed (Fig. 5a and Supplementary Fig. 22), indicating that CO_2_ reduction to CO occurred through route (i). Namely, [Cu(H_2_O)]^+^ first interacted with CO_2_ by a formation of [Cu(H_2_O)(CO_2_)]^+^, which was supported by isotope-labeling experiments (Supplementary Fig. 24), and then a reduction reaction occurred to convert CO_2_ to CO. The above results also indicated that the coordinated H_2_O on the Cu^+^, rather than free H_2_O, played crucial roles for the reduction of CO_2_ to CO. The transition metals such as Cu^+^ not only provided efficient active sites for the generation of [Cu(H_2_O)]^+^ or other H_2_O-based metal complex ions, but also offered opportunity to charge neutral species such as CO_2_ and CO for favorable mass spectrometric analysis by the forms of [Cu(CO_2_)]^+^ and [Cu(CO)]^+^ (Figs. 2 and 4).

Based on the prior experiments, the origin of the O atom in CO was from H_2_O rather than CO_2_. Thus, the two O atoms in CO_2_ needed to be eliminated prior to the formation of CO. According to the previous CO_2_RR mechanisms^46,48,70^, the generation of CO was achieved by first proton-electron (H^+^/e^−^) transfer processes to CO_2_, followed by the elimination of H_2_O. To promote the formation of CO, an efficient step to proton generation and capture was necessary^46^. In this study, a proton (H^+^) was also found to be crucial to the generation of CO. For the reaction of [Cu(H_2_O)]^+^ and CO_2_, the generation of H^+^ was believed to be caused by water dissociation. However, further dissociation of water might affect its ability to participate in the formation of [Cu(H_2_O)(CO_2_)]^+^ and reduce the reaction efficiency. Addition of supplementary H^+^ could mitigate water dissociation and boost CO generation. To confirm this hypothesis, 2 μL of acetic acid (HOAc) was injected into the CO_2_ gas circuit system. For the system containing [Cu(H_2_O)]^+^, a 8.9-fold increase of CO formation was observed with the addition of HOAc, whereas no significant difference was observed for bare Cu^+^ (Fig. 5b and Supplementary Figs. 25 and 26). These results indicated that supplementary H^+^ was indeed important for boosting the reduction efficiency of CO_2_ to CO in the presence of [Cu(H_2_O)]^+^ catalyst, in which H^+^ from the coordinated H_2_O participated into the elimination of O atoms in CO_2_^46,48,70^.

Many previous studies^8,26,55–59,74,75^ illustrated that the Cu(I) surface was the active site anchoring the CO_2_RR. Because of this, several strategies, including the addition of oxygen^59^ or copper nitride support^76^, boron-element doping^39^, plasma treatment^77^, catalyst electro-redeposition^78^, and covering the catalyst surface with nanocavities^55^, have been used to stabilize the active Cu(I) oxidation state. Despite these efforts, the precise effect of a single Cu valence state on the reduction of CO_2_ to CO remains ambiguous. Ions can be easily isolated through MS, enabling the investigation of the interaction between CO_2_ and Cu with different oxidation states. In this study, we not only investigated the effect of Cu(I) in CO_2_RR, but also explored the performance of Cu(II) on CO production. Taking the copper oxidation state and the aforementioned H_2_O effect into account, [^63^Cu(OH)·H_2_O]^+^ and [^65^Cu(OH)·H_2_O]^+^ (Supplementary Fig. 27a, b) were isolated to interact with CO_2_. However, in contrast to the Cu(I) system (Fig. 4), a much lower amount of [^63^Cu(CO)]^+^ or [^65^Cu(CO)]^+^ was observed in the mass spectrum (Supplementary Fig. 27c, d). These results indicated that Cu(I) was better at reducing CO_2_ to CO than Cu(II) even in the presence of water, which was in good agreement with the above discussion.

### Mechanistic studies

**Fig. 6:**
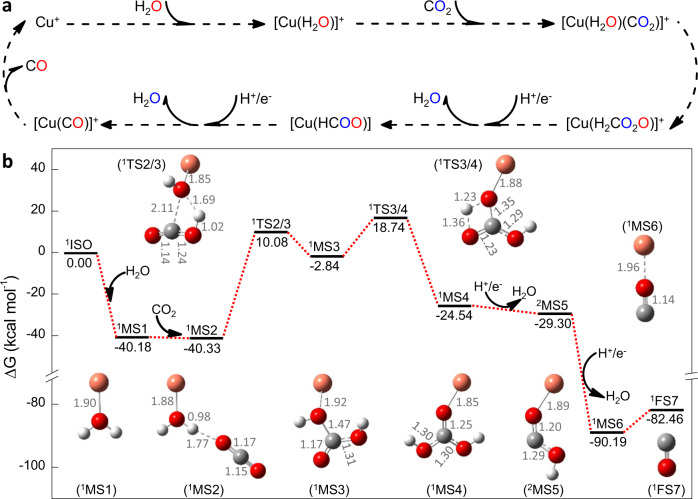
The mechanism of CO_2_ reduction to CO in the presence of Cu(I) catalyst and water. **a** Schematic representation of the reduction of CO_2_ to CO according to experimental observations. **b** The DFT microscopic reaction pathway [the singlet reactants of ^1^Cu^+^ + ^1^H_2_O + ^1^CO_2_ (^1^IS0, 0.00) was taken to be zero as a reference], demonstrating the thermodynamic and kinetic feasibility of the suggested pathway.

Based on the overwhelming evidence from the experimental observations, the pathway of CO_2_ reduction to CO over Cu(I)-based catalyst was suggested in Fig. 6a. This process started with the formation of [Cu(H_2_O)]^+^, which interacted with CO_2_ to generate [Cu(H_2_O)(CO_2_)]^+^. As aforementioned, H_2_CO_3_ formed after the reaction of CO_2_ and H_2_O, thereby the occurrence of transition from [Cu(H_2_O)(CO_2_)]^+^ to [Cu(H_2_CO_3_)]^+^, which was similar to the direct CO_2_ capture and conversion to fuels over magnesium nanoparticles^54^. Subsequent H^+^/e^−^ transfer reactions^46,48,70^ led to the generation of [Cu(CO)]^+^, along with the elimination of two H_2_O molecules. In the CO_2_RR, H^+^ ions originated from the dissociation of H_2_O/H_2_CO_3_ or added acid, and the necessary electrons involved in the reduction of CO_2_ to CO were likely from the generated H_2_CO_3_ (Supplementary Fig. 28 and related discussion). Upon the CO_2_ reduction, the O atom in [Cu(H_2_O)]^+^ combined with the C atom in CO_2_ by the formation of CO, whereas the two O atoms in CO_2_ were eliminated by loss of two H_2_O molecules. The released H_2_O molecules could combine with Cu^+^ to form [Cu(H_2_O)]^+^ for the next cycle of CO_2_ reduction. Despite this, the oxidation state of copper persisted +1, in good agreement with the previous report ^77^.

To gain detailed insight into the reaction mechanism, DFT calculations were carried out to explore the key reaction intermediates and possible reaction pathways. Both the singlet and triplet reaction paths had been considered to understand all possibilities. The singlet reaction path was more thermodynamically favorable than the triplet reaction path, and both paths were similar (Fig. 6b, Supplementary Fig. 29, and Supplementary Table 2–4). Because of this fact, the discussion herein focused mainly on the singlet reaction path with a lower energy.

The activation of CO_2_ was initialized with the collision interaction among ^1^CO_2_, ^1^Cu^+^, and ^1^H_2_O (Supplementary Fig. 30). All calculations on bimolecular interactions indicated that ^1^[Cu-OH_2_]^+^ (^1^MS1), through the collision of ^1^Cu^+^ and ^1^H_2_O, was the most preferred possibility in contrast to others, such as ^1^Cu^+^ and ^1^CO_2_ or ^1^CO_2_ and ^1^H_2_O. The binding energy of ^1^Cu^+^ and ^1^H_2_O in ^1^MS1 was as high as −40.18 kcal mol^−1^, which was larger than those of ^1^Cu^+^ and ^1^CO_2_ (^1^[Cu-O = C = O]^+^, −19.68 kcal mol^−1^) and ^1^CO_2_ and ^1^H_2_O (^1^[H_2_O-O = C = O]^+^, 2.53 kcal mol^−1^). In the collision between ^1^Cu^+^ and ^1^H_2_O, ^1^Cu^+^ was prone to interact with the central O atom in H_2_O to form ^1^[Cu-OH_2_]^+^ through O-Cu coupling with a bond length of 1.90 Å.

The existence of activated ^1^[Cu-OH_2_]^+^ (^1^MS1) promoted the reduction of ^1^CO_2_. First, ^1^CO_2_ interacted with ^1^MS1 to form the ^1^[Cu-O(H)-H···OCO]^+^ (^1^MS2, −40.33 kcal mol^−1^) intermediate by a weak O-H···O hydrogen bond. The O-C bond length of ^1^CO_2_ changed from 1.16 Å to 1.17 and 1.15 Å in ^1^MS2. Subsequently, ^1^MS2 isomerized into ^1^[Cu-O(H)-C(O)OH]^+^ (^1^MS3, −2.84 kcal mol^−1^) through a transition state, ^1^TS2/3 (10.08 kcal mol^−1^). It is worth noting that in the conversion from ^1^TS2/3 to ^1^MS3, the breakage of the O-H bond in H_2_O occurred, and the O-H and C-O bonds formed between the H_2_O and CO_2_ (Supplementary Fig. 31–34). This process was the rate-limiting step of CO_2_ reduction with an activation barrier as high as 50.41 kcal mol^−1^. Further isomerization turned ^1^MS3 into ^1^[Cu-O-C(OH)_2_]^+^ (^1^MS4, −2.84 kcal mol^−1^), which only involved the migration of intramolecular hydrogen through the transition state ^1^TS3/4 (18.74 kcal mol^−1^), and the activation barrier from ^1^MS3 to ^1^TS3/4 was 21.58 kcal mol^−1^.

Once ^1^MS4 was generated, two molecules of water were lost by a H^+^/e^−^ transfer process, thereby leading to the formation of ^1^[Cu-O-C]^+^ (^1^MS6, −90.19 kcal mol^−1^). In the process, H^+^/e^−^ was initially transferred from the reaction system to the OH group of ^1^MS4, which resulted in the release of one H_2_O molecule to form a ^1^[Cu-O-COH]^+^ (^2^MS5, −29.30 kcal mol^−1^) intermediate. As the OH group in ^2^MS5 was further attacked by H^+^/e^−^, a ^1^MS6 intermediate was formed through the release of another H_2_O molecule. The dehydration process would compete with hydrogen evolution reaction (HER). By calculation of their corresponding free energy, the values were −4.76 kal mol^−1^ and −60.89 kal mol^−1^ when ^1^MS4 and ^2^MS5 were transferred to ^2^MS5 and ^1^MS6, respectively. For a standard hydrogen electrode, the free energy is −19.09 kal mol^−1^ as H^+^ is transferred to H_2_ in basic solution, which system is favorable to aqueous electrochemical CO_2_RR while preventing from HER. Apparently, in contrast to the transfer of ^1^MS4 to ^2^MS5, it was prone to the HER, whereas an opposite trend occurred when ^2^MS5 was transferred to ^1^MS6. Along with the processes, ^2^MS5 would also tend to combine with H^+^ by formation of [Cu(HCOOH)]^+^ as a side reaction, which was captured in our current study (Supplementary Figs. 7a, b and 35).

In the last step, the bound CO group dissociated from ^1^MS6 by forming an isolated CO molecule and pristine ^1^Cu^+^, thus completing the catalytic cycle and releasing −82.46 kcal mol^−1^ of thermal energy. The reaction pathway offers further convincing theoretical evidence for the hypothesized mechanism derived from mass spectrometric analysis. It also details how H_2_O molecule is involved in the reduction of CO_2_ to CO and how it replaces two O atoms in CO_2_ with the one in its structure.

In summary, we have developed a modified TSQ mass spectrometer that enabled online observation of the CO_2_RR and detection of the reaction intermediates and products. The results demonstrated that the coordinated H_2_O on Cu(I)-based catalysts played a crucial role in the efficiencies of both CO_2_ adsorption onto the Cu(I) catalyst and CO_2_ reduction to CO, and that an improvement of two orders of magnitude was achieved in the presence of a coordinated H_2_O than without H_2_O. Further experiments indicated that the existing form of H_2_O and the number of coordinated H_2_O also had a significant effect on the reduction of CO_2_ to CO. More importantly, isotope-labeling investigations revealed that the origin of the O atom in the generated CO originated from H_2_O, instead of CO_2_. Based on the experimental observations and computational calculations, the specific pathway for the reduction of CO_2_ to CO was proposed. This work not only offers a new strategy to disclose the reaction process of CO_2_RR, but also provides useful insight into the roles of H_2_O, suggesting the efficiency of CO_2_RR could be improved by constructing new types of catalysts with coordinated H_2_O.

## Methods

### Preparation of different Cu-based solutions

Identical procedures were used to prepare the different Cu-based solutions. Specifically, 0.1 g of Cu-based particles such as CuCl were first dissolved into 1.0 mL of double-deionized water. After sonication for 30 min using a KQ3200DB ultrasonic cleaner (Kunshan Ultrasonic Instrument Co., Ltd., Kunshan, China), 1 μL of the CuCl aqueous solution was added into 999 μL of acetonitrile. Finally, the resulting solution was mixed with a QL-901 Vortex oscillator (Haimen Qilin Beier Instrument Manufacturing Co., Ltd., Haimen, China) for 1 min.

### Preparation of glass capillary with a tip orifice of 1 μm

A glass capillary with a tip orifice of 1 μm was pulled from borosilicate glass capillary with filament (Sutter Instrument, USA, 1.5 mm o.d., 0.86 mm i.d., 10 cm length) using a micropipette puller (Model P-97, Sutter Instrument Co., Novato. CA, USA). The tip orifice was measured with a metallographic microscope equipped with a DCA 10.0 digital camera (1 million resolution) and had a tip orifice precision of ± 1 μm.

### Online reaction and MS analysis

All experiments on nanoelectrospray ionization mass spectrometry (nanoESI-MS) were carried out with either a TSQ Quantum Access Max mass spectrometer or an Orbitrap Elite Hybrid Ion Trap-Orbitrap Mass Spectrometer (Thermo Fisher Scientific, San Jose, CA, USA). For nanoESI, 20 μL of a 100 μg mL^−1^ CuCl solution was injected into a glass capillary with 1 μm of tip orifice, and, subsequently, 1.0 kV of DC voltage was applied to the CuCl solution for the generation of various Cu-based ions. The distance between the nanoESI tip and MS inlet capillary was about 10 mm. Mass spectra were recorded in the positive ion mode with a capillary temperature of 270 °C. The identification of Cu-based ions was confirmed by high-resolution mass spectrometry (HRMS) and tandem mass spectrometry (MS/MS) using collision-induced dissociation (CID). Argon gas (99.995% purity) was used as the collision gas. For the reaction between Cu-based ions and CO_2_, Cu-based ions such as Cu^+^ and [Cu(H_2_O)]^+^ were first isolated from Q1 and then introduced to Q2 (see Fig. 1). In the reaction unit of Q2, Cu-based ions interacted with CO_2_ to yield CO under collision energy of 5 V, and the resulting products were subsequently transferred to Q3, followed by detection. It should be pointed out that the collision energy for CO_2_ reduction was controlled by the operation software of employed commercial mass spectrometer. To explore the effect of acid on the reaction of Cu-based ions and CO_2_, 2 μL of acetic acid was injected through a six-way valve (Beijing Yijia Technology Co., Ltd., Beijing, China) into the CO_2_/Ar gas circuit system. To enhance the reaction efficiency between Cu-based ions and CO_2_, the gas circuit system was heated to different temperatures via a heating tape controlled by an XMT-815 temperature controller (Shanghai Mike Instrument Co., Ltd., Shanghai, China). The actual temperature of heating tape was determined using a 17B Fluke multimeter (Shanghai Fluke Corporation, Shanghai, China).

### Reversed WGSR experiment

The experiments were carried out with a WGSR apparatus (Tianjin Tongyuan Hengli Technology Co., Ltd, Tianjin, China). The reactor was loaded with 1.0 g of Cu/ZnO/Al_2_O_3_ catalyst. The pressures of H_2_^16^O and H_2_^18^O were controlled by a syringe pump with flow rates ranging from 1 to 50 μL min^−1^. After adjusting a flow rate, a balance time of 1.5 h was used to make the equilibrium of CO_2_ reduction reaction. The air and involved contaminants were replaced with H_2_ via successive purging. In the reaction process, the reaction partial pressure of H_2_ and CO_2_ were kept constant at 2.7 MPa and 0.3 MPa, respectively, and the reactor temperature was maintained at 230 ^o^C. The gas products were analyzed online with a Fuli GC9790 gas chromatograph equipped with a TDX-01 molecular sieve packing column (2 m x 3 mm). For the H_2_^18^O-labelling experiments, the collected gas products were analyzed off-line with an Agilent Technologies 7890B gas chromatograph equipped with a GS-CarbonPLOT capillary column (30 m x 0.32 mm I.D., 1.50 μm film thickness) and an Agilent Technologies 5977 A mass spectrometer. The carrier gas was helium with a flow rate of 15 mL min^−1^. The initial oven temperature was set as 35 °C and maintained for 3 min. Afterward, it was increased to 100 °C with a rate of 40 °C min^−1^ followed by maintaining for 1 min. The injector temperature was 185 °C, and the injection volume was 250 μL with a split ratio of 100:1.

### In situ DRIFTS analysis

The in situ diffuse reflectance infrared Fourier transform spectroscopy (in situ DRIFTS) results were conducted on an FTIR (Nicolet 6700) equipped with a Pike DRIFT cell (PIKE Technologies) with a KBr window and an MCT/A detector (cooled by liquid nitrogen). The spectra were collected in the range from 900 to 4000 cm^−1^ with 32 scans, and the resolution was 4 cm^−1^. All used cylinder gases were of high purity and dried through a moisture trap (Agilent Technologies) before entering the in situ chamber. To minimize the environment interference, the FTIR chamber was purged with argon (99.999%) in a flow rate of 4 L min^−1^. Prior to the experiment, the sample was treated with hydrogen (99.9999%) in a flow rate of 5 mL min^−1^ for 2 h and then switched to helium (99.9999%) with a flow rate of 5 mL min^−1^ for 2 h at 500 °C to reduce the surface oxide and remove all possible organic contaminants. Subsequently, under the helium flow, the reactor was cooled down to 150 °C or 250 °C for hydrogenation of CO_2_. When a steady baseline was obtained, hydrogen (3 mL min^−1^) and CO_2_ (4 mL min^−1^, 5% CO_2_/95% Ar) were introduced sequentially. To investigate the effect of water on CO_2_RR, a syringe needle fulfilled with 1 μL of water was inserted into the tube containing hydrogen flow, enabling the introduction of trace water into the reaction system. The employed catalyst, Cu/γ-Al_2_O_3_, was prepared by an incipient-wetness impregnation method using the precursors of Cu(NO_3_)_2_ (>99.0%, Guangzhou Chemical Reagent Factory, Guangzhou, China) and γ-Al_2_O_3_ powder (99.99%, Energy Chemical Co., Guangzhou, China).

### Electrochemical reduction of CO_2_ and online mass spectrometer monitoring

For the electrochemical reduction of CO_2_ to CO, the reaction products and CO_2_ were monitored with an online differential electrochemical mass spectrometer (DEMS, Linglu Instrument Co., Ltd, Shanghai, China). The configuration of the electrochemical cell is shown in Supplementary Fig. 18. The cell was fulfilled with 2.5 mL of 0.5 M KCl solution, which was saturated with CO_2_ gas. During the electrochemical test, a continuous CO_2_ gas flow was introduced into KCl solution. The working electrode was prepared by sputtering gold nanoparticles on porous membrane. The reference electrode was an Ag/AgCl electrode in saturated KCl solution, and the counter electrode was Pt wire electrode. The linear sweeping voltammetry was conducted on a electrochemical workstation (CHI Instruments, Inc., Austin, TX, USA) with the sweeping rate of 10 mV s^−1^ from −0.8 V to −1.6 V. Simultaneously, the MS signals with the mass/charge ratios of 4430, and 28 were recorded by the MS. The ^18^O-labeled H_2_O (purity: 99.0%, ^18^O abundance: ≥98.0%) was purchased from Wuhan Isotope Technology Co., Ltd (Wuhan, China).

### Computational details

All electronic structure calculations were performed with the Gaussian 09 package (revision B.01; Gaussian, Inc., Wallingford CT, 2010)^79^. For geometry optimization, we used the B2PLYP double hybrid density functional method^80^ in conjunction with the augmented correlation-consistent polarized triple zeta (aug-cc-pVTZ) basis set with the implicit treatment of scalar-relativistic effects by using the effective core potential (ECP) pseudopotential for the metal atoms^81^, and the aug-cc-pVTZ all-electron basis set for all other atoms^82^. Harmonic vibrational frequencies were computed to verify the nature of the stationary points. The minimum structures reported in this work showed only positive eigenvalues of the Hessian matrix, whereas the transition states had one negative eigenvalue. Intrinsic reaction coordinate calculations were also performed to confirm that the transition states were correlated with the designated intermediates^83–86^. The zero-point vibrational energy (ZPVE) and thermal corrections to the enthalpy were calculated for structures optimized at the B2PLYP/cc-pVTZ level. The thermodynamic functions (ΔH) were estimated within the ideal gas, rigid-rotor, and harmonic oscillator approximations at 298 K and 1 atm. For ease of discussion, the symbols “^S^IS” and “^S^FS” are used to describe the initial state (IS), intermediate state (MS), and final state (FS), while “^S^TSm/n” is used for the interconversion transition state between the intermediate states, ^S^m and ^S^n. The left superscript “S” denotes the spin multiplicity (1 and 3 for singlet and triplet, respectively).

## Supplementary information

Supplementary Information

Peer Review File

## Data Availability

Source data are provided with this paper, which can also be available from the corresponding authors on reasonable request.

## Source data

Source Data
