# Supplementary material for: Water coordinated on Cu(I)-based catalysts is the oxygen source in CO2 reduction to CO
Source: Nat Commun. 2022 May 11;13:2577. doi: 10.1038/s41467-022-30289-5 (PMC9095693; doi:10.1038/s41467-022-30289-5)
Supplement: Supplementary file 1 — Supplementary Information [file 41467_2022_30289_MOESM1_ESM.pdf]

Supplementary Information for

Water Coordinated on Cu(I)-based Catalysts is the Oxygen Source in CO_2 Reduction to CO

Yajun Zheng[1†], Hedan Yao[1†], Ruinan Di[2†], Zhicheng Xiang[1], Qiang Wang[2*], Fangfang Lu[1], Yu Li[1], Guangxing Yang[3*], Qiang Ma[4], and Zhiping Zhang[1*]

[1] School of Chemistry and Chemical Engineering, Xi'an Shiyou University, Xi'an 710065, China

[2] School of Chemistry and Molecular Engineering, Nanjing Tech University, Nanjing 211816, China

[3] School of Chemistry and Chemical Engineering, South China University of Technology, Guangzhou, 510641, China

[4] Chinese Academy of Inspection and Quarantine, Beijing 100176, China

[†] These authors contributed equally: Yajun Zheng, Hedan Yao, Ruinan Di.

Included materials:

Supplementary Note 1: Chemicals and materials

Supplementary Figure 1: Full mass spectra of different Cu-based solutions

Supplementary Figure 2: Full mass spectra of CuCl solutions by dissolving it into different solvents

Supplementary Figure 3: Experimental data and theoretical value of some peaks using a high resolution Orbitrap mass spectrometer

Supplementary Figure 4: Mass spectra of reduction of CO_2 to CO under different Ag-based catalytic systems

Supplementary Figure 5: ass spectra of reduction of CO_2 to CO under different Pd-based catalytic systems

Supplementary Figure 6: Effect of H_2O pressure on the reduction of CO_2 to CO using a water-gas shift reaction (WGSR) apparatus equipped with gas chromatography

Supplementary Figure 7: *In situ* DRIFT spectra of the resulting products on Cu/γ-Al_2O_3 or Pt/γ-Al_2O_3 with addition of H_2O into the reaction system of CO_2 and H_2 at different reaction temperatures

Supplementary Figure 8: Variation of CO_2 adsorption and CO generation with reaction pressure and temperature of heating tape around the gas circuit

Supplementary Figure 9: Interaction of methanol (CH_3OH, M.W. = 32) and different Cu-based species

Supplementary Figure 10: Interaction of ethanol (CH_3CH_2OH, M.W. = 46) and different Cu-based species

Supplementary Figure 11: Interaction of acetonitrile (CH_3CN, M.W. = 41) and different Cu-based species

Supplementary Figure 12: Interaction of benzene (C_6H_6, M.W. = 78) and different Cu-based species

Supplementary Figure 13: Interaction of toluene (C_7H_8, M.W. = 92) and different Cu-based species

Supplementary Figure 14: Interaction of dichloromethane ($CH_2{}^{35}Cl_2$, M.W. = 84; $CH_2{}^{35}Cl^{37}Cl$, M.W. = 86) and different Cu-based species

Supplementary Figure 15: Variation in the peak intensity of $[^{63}Cu(H_2O)]^+$ with reaction pressures and gas circuit temperatures

Supplementary Figure 16: Isotope-labeling MS measurement results under different ^{65}Cu-based reaction systems

Supplementary Figure 17: Isotope-labeling MS measurement results under different $^{107}Ag/^{104}Pd$-based reaction systems

Supplementary Figure 18: Differential electrochemical mass spectrometer for the electrochemical reduction of CO_2 to CO and *in situ* mass spectrometer monitoring of resulting reaction products

Supplementary Figure 19: Simultaneous measurement of the MS current of different ions using an *in situ* differential electrochemical mass spectrometer (Au electrode)

Supplementary Figure 20: Simultaneous measurement of the MS current of different ions using an *in situ* differential electrochemical mass spectrometer (Ag electrode)

Supplementary Figure 21: Simultaneous measurement of the MS current of different ions using an *in situ* differential electrochemical mass spectrometer (Pd electrode)

Supplementary Figure 22: Effects of coordinated H_2O number ($^{65}Cu^+$, $[^{65}Cu(H_2O)]^+$ and $[^{65}Cu(H_2O)_2]^+$) and free H_2O on the reduction of CO_2 to CO under different Cu-based catalytic systems

Supplementary Figure 23: Mass spectra of the different systems after interaction between free $H_2{}^{18}O$ and CO_2

Supplementary Figure 24: Mass spectra of the different reaction systems for capturing $[Cu(CO_2)(H_2O)]^+$ intermediates

Supplementary Figure 25: Mass spectra of the different reaction systems without and with HOAc

Supplementary Figure 26: Influence of the extraneous acid on the generation of CO

Supplementary Figure 27: MS/MS spectra of $[^{63}Cu(OH)(H_2O)]^+$ and $[^{65}Cu(OH)(H_2O)]^+$ and their catalysis effects to CO_2 reduction

Supplementary Figure 28: Mass spectra of the products by interaction between $[Cu(H_2O)]^+$ and CO_2 and variation in the peak intensity of different Cu-based species with increasing collision energy

Supplementary Figure 29: Reaction pathways of CO_2 reduction to CO catalyzed by Cu^+ and H_2O

Supplementary Figure 30: Optimal geometric structures and interaction energies among $^1CO_2$, $^1Cu^+$ and ^1H_2O at the B2PLYP/cc-PVTZ/Aug-cc-pVTZ-PP level

Supplementary Figure 31: Schematic diagram of the bond breaking/making in the single step along IRC calculations of the ^{1}TS2/3 at the B2PLYP/cc-PVTZ/Aug-cc-pVTZ-PP level

Supplementary Figure 32: Schematic diagram of the bond breaking/making in the single step along IRC calculations of the ^{1}TS3/4 at the B2PLYP/cc-PVTZ/Aug-cc-pVTZ-PP level

Supplementary Figure 33: Optimal geometric structure of singlet intermediates and transition states of the reduction of CO_2 to CO catalyzed by on Cu^+ and H_2O at the B2PLYP/cc-PVTZ/Aug-cc-pVTZ-PP level. Bond lengths are in angstroms and angles in degrees

Supplementary Figure 34: Optimal geometric structure of triplet intermediates and transition states of the reduction of CO_2 to CO catalyzed by on Cu^+ and H_2O at the B2PLYP/cc-PVTZ/Aug-cc-pVTZ-PP level. Bond lengths are in angstroms and angles in degrees

Supplementary Figure 35: Mass spectra of CO_2 reduction to CO under different Cu-based catalytic systems

Supplementary Table 1: Comparison of the reaction conditions among TSQ mass spectrometer, realistic thermal (reverse water-gas shift reaction) and electrochemical catalysis

Supplementary Table 2: Total (a.u.) and relative (kcal/mol) energies of the reaction species at B2PLYP/cc-pVTZ/Aug-cc-pVTZ-PP levels with inclusion of B2PLYP/cc-pVTZ/Aug-cc-pVTZ-PP zero-point vibrational energies (ZPVE)

Supplementary Table 3: Total (a.u.) and relative (kcal/mol) energies of the reaction species and transition states for CO_2 reduction catalyzed by singlet Cu^+ at B2PLYP/cc-PVTZ/Aug-cc-pVTZ-PP levels with inclusion of B2PLYP/cc-PVTZ/Aug-cc-pVTZ-PP zero-point vibrational energies (ZPVE)

Supplementary Table 4: Total (a.u.) and relative (kcal/mol) energies of the reaction species and transition states for CO_2 reduction catalyzed by triplet Cu^+ at B2PLYP/cc-PVTZ/Aug-cc-pVTZ-PP levels with inclusion of B2PLYP/cc-PVTZ/Aug-cc-pVTZ-PP zero-point vibrational energies (ZPVE)

Supplementary References: References 1-9

Supplementary Note 1: Chemicals and materials

All chemicals employed were from commercially available sources. Both Cu (60 nm) and CuO (50 nm) particles were ordered from Shanghai Chaowei Nano Technology Co., Ltd (Shanghai, China), and Cu_2O (20 nm) particles were from Tianjin Institute of Guangfu Fine Chemical Industry (Tianjin, China). CuCl, CuBr and CuI were purchased from Tianjin Beilian Fine Chemicals Development Co., Ltd. (Tianjin, China), Shanghai Zhenxin Reagent Factory (Shanghai, China), and Chengdu Kelong Chemical Reagent Factory, respectively. Both acetonitrile and ethanol were ordered from Beijing J&K Scientific Ltd. (Beijing, China), methanol, tert-butanol, tetrahydrofuran and dimethyl sulfoxide were from Thermo Fisher Scientific (New Jersey, USA), Tianjin Hedong Hongyan Reagent Factory (Tianjin, China), Tianjin Kemiou Chemical Reagent Co., Ltd. (Tianjin, China) and Tianjin No.1 Chemical Reagent Factory (Tianjin, China), respectively. Dichloromethane, benzene and toluene were purchased from Sweden Oceanpak Company (Sweden), Tianjin Fuyu Fine Chemical Co., Ltd. (Tianjin, China) and Luoyang Haohua Chemical Reagent Co., Ltd. (Luoyang, China). Acetic acid was ordered from Beijing J&K Scientific Ltd. (Beijing, China). $H_2{}^{16}O$ was double-deionized water generated from ULUPURE ultrapure water treatment equipment (Xi'an ULUPURE Instrument Equipment Co., Ltd., Xi’an, China), and $H_2{}^{18}O$ (purity: ≥99.0%, ^{18}O abundance: ≥98.0%) was purchased from Shanghai Research Institute of Chemical Industry Co., Ltd. (Shanghai, China). The mixture gases [5% $C^{16}O_2$ (purity: 99.99%) and 95% argon (purity: 99.99%) 5% $C^{18}O_2$ (purity: 99.0%, ^{18}O abundance: ≥98.0%) and 95% argon (purity: 99.99%)] were ordered from Xi'an Tenglong Chemical Co., Ltd. (Xi’an, China). All chemicals and reagents were of analytical grade or a better grade and used without further purification.

Supplementary Figures 1-35:

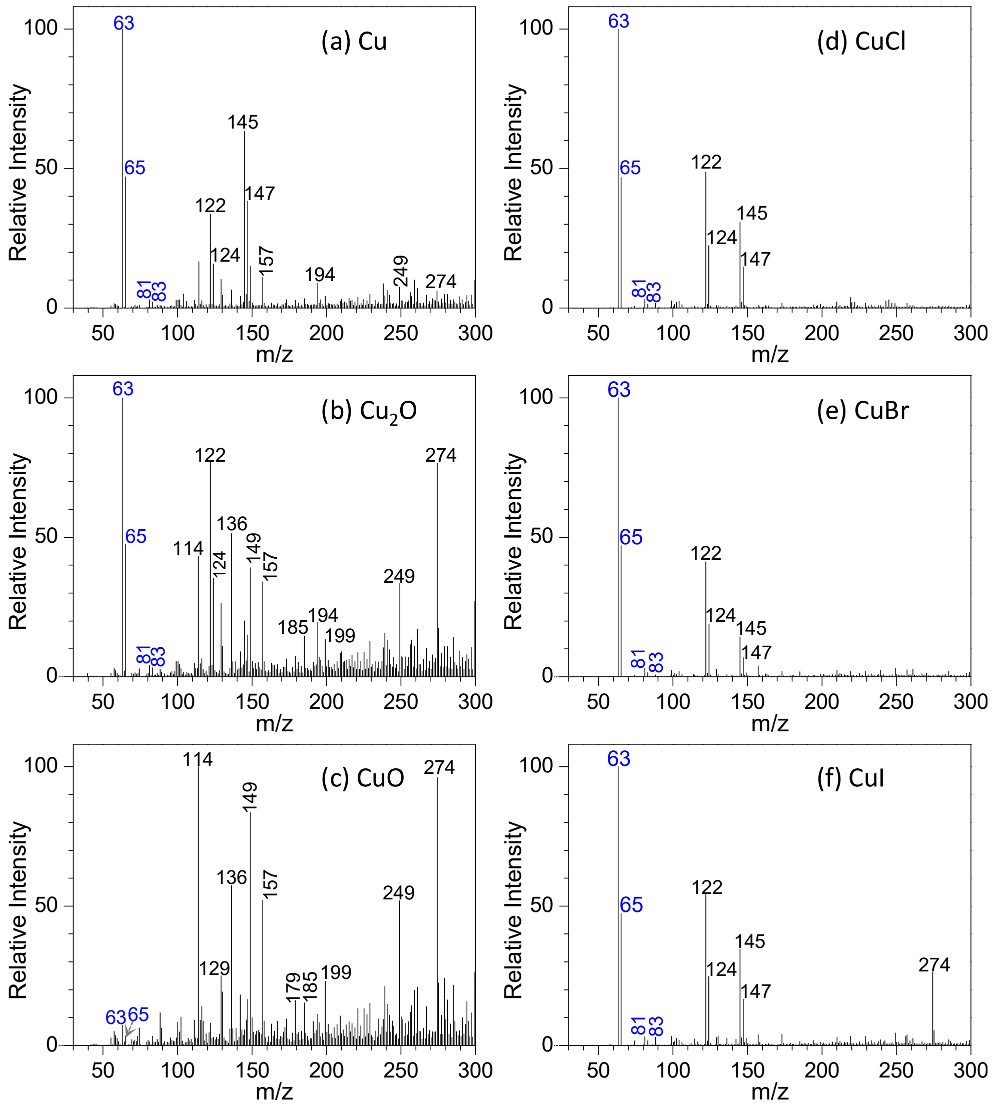

Supplementary Figure 1 | Full mass spectra of different Cu-based solutions: **(a)** Cu, **(b)** Cu_2O, **(c)** CuO, **(d)** CuCl, **(e)** CuBr, and **(f)** CuI (concentration of different Cu-based species in acetonitrile: 100 μg mL^{-1}; spray voltage: 1.0 kV).

Note: m/z 122, 124, 145 and 147 in the mass spectra could be assigned as $[^{63}Cu(CH_3CN)(H_2O)]^+$, $[^{65}Cu(CH_3CN)(H_2O)]^+$, $[^{63}Cu(CH_3CN)_2]^+$, and $[^{65}Cu(CH_3CN)_2]^+$, respectively.

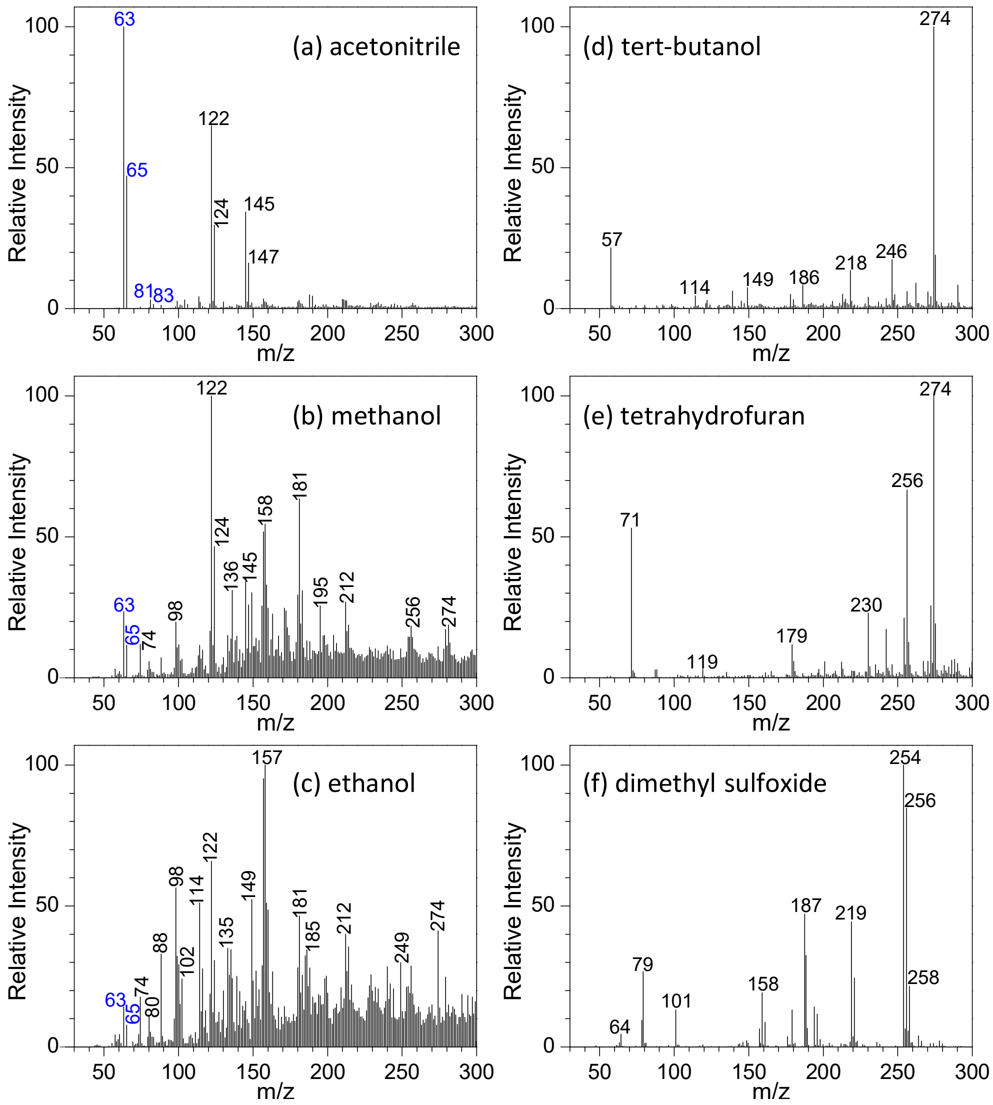

Supplementary Figure 2 | Full mass spectra of CuCl solutions by dissolving it into different solvents: **(a)** acetonitrile, **(b)** methanol, **(c)** ethanol, **(d)** tert-butanol, **(e)** tetrahydrofuran, and **(f)** dimethyl sulfoxide (CuCl concentration: 100 μg mL^{-1}; spray voltage: 1.0 kV).

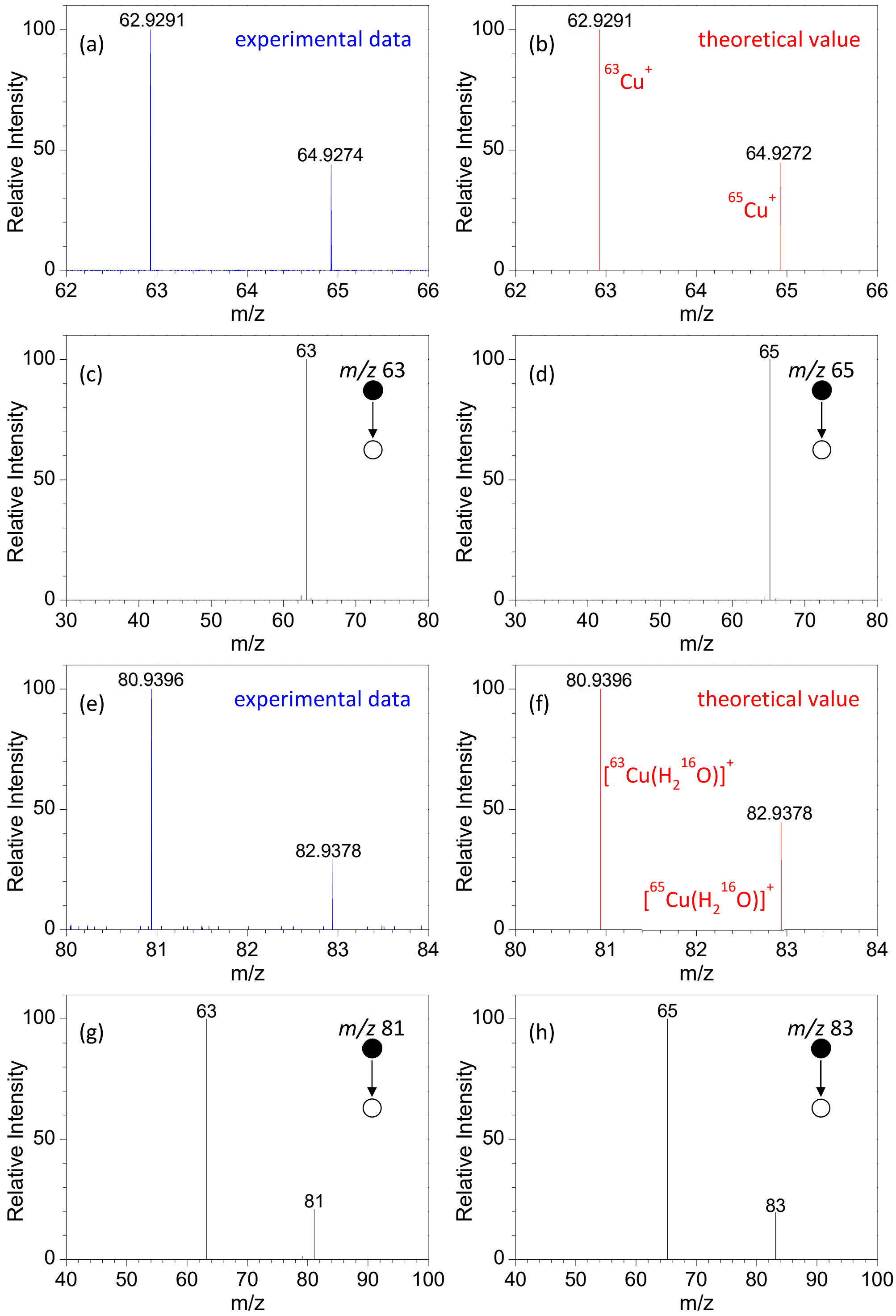

Supplementary Figure 3 | Experimental data and theoretical value of some peaks using a high resolution Orbitrap mass spectrometer: **(a)** and **(b)** m/z 62.9291 ($^{63}Cu^+$) and m/z 64.9274 ($^{65}Cu^+$); MS/MS spectra for **(c)** m/z 63 and **(d)** m/z 65; Experimental data and theoretical value of some peaks using a high resolution Orbitrap mass spectrometer: **(e)** and **(f)** m/z 80.9396 ($[^{63}Cu(H_2{}^{16}O)]^+$) and m/z 64.9274 ($[^{65}Cu(H_2{}^{16}O)]^+$); MS/MS spectra for **(g)** m/z 81 and **(h)** m/z 83 (sample: acetonitrile solution containing 100 μg mL^{-1} CuCl; spray voltage: 1.0 kV).

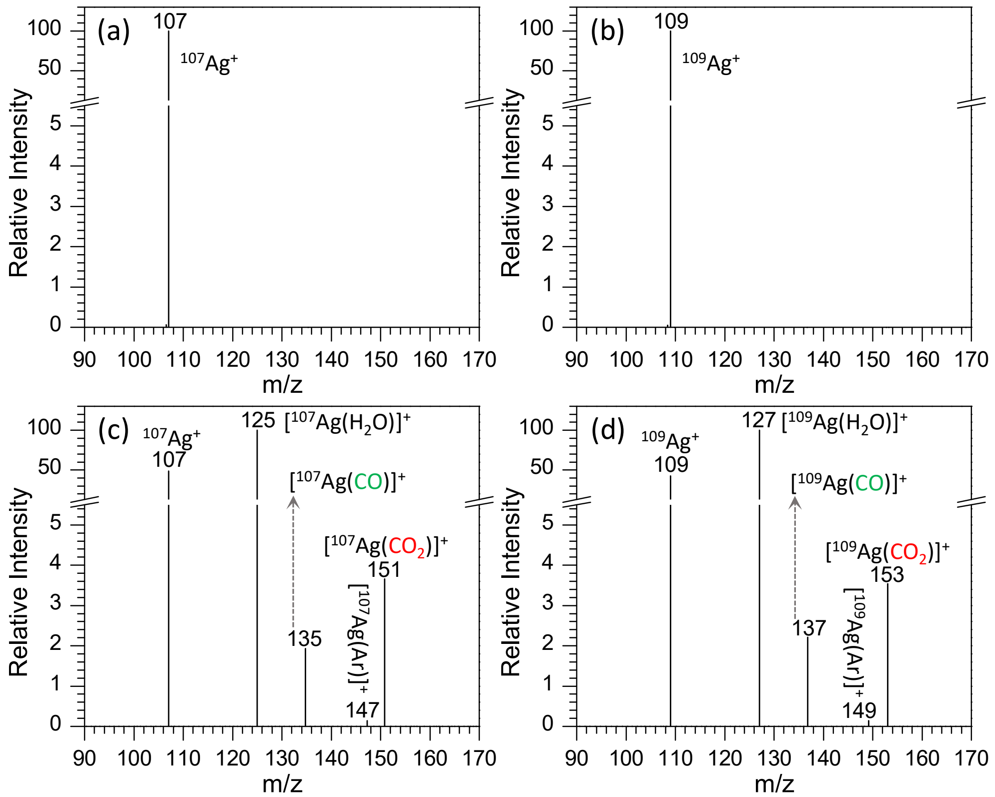

Supplementary Figure 4 | Mass spectra of reduction of CO_2 to CO under different Ag-based catalytic systems: **(a)** $^{107}Ag^+$, **(b)** $^{109}Ag^+$, **(c)** $[^{107}Ag(H_2O)]^+$, and **(d)** $[^{109}Ag(H_2O)]^+$ (gas circuit temperature: 280 ^{o}C; reaction pressure: 1.5 mTorr).

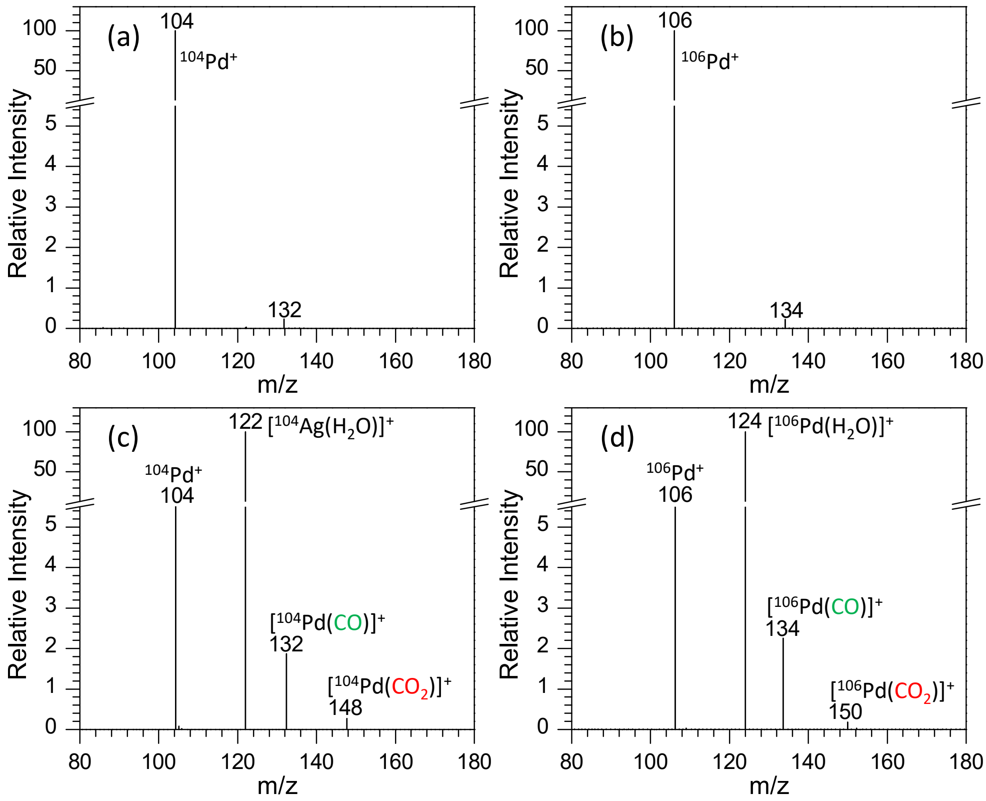

Supplementary Figure 5 | Mass spectra of reduction of CO_2 to CO under different Pd-based catalytic systems: **(a)** $^{104}Pd^+$, **(b)** $^{106}Pd^+$, **(c)** $[^{104}Pd(H_2O)]^+$, and **(d)** $[^{106}Pd(H_2O)]^+$ (gas circuit temperature: 280 ^oC; reaction pressure: 1.5 mTorr).

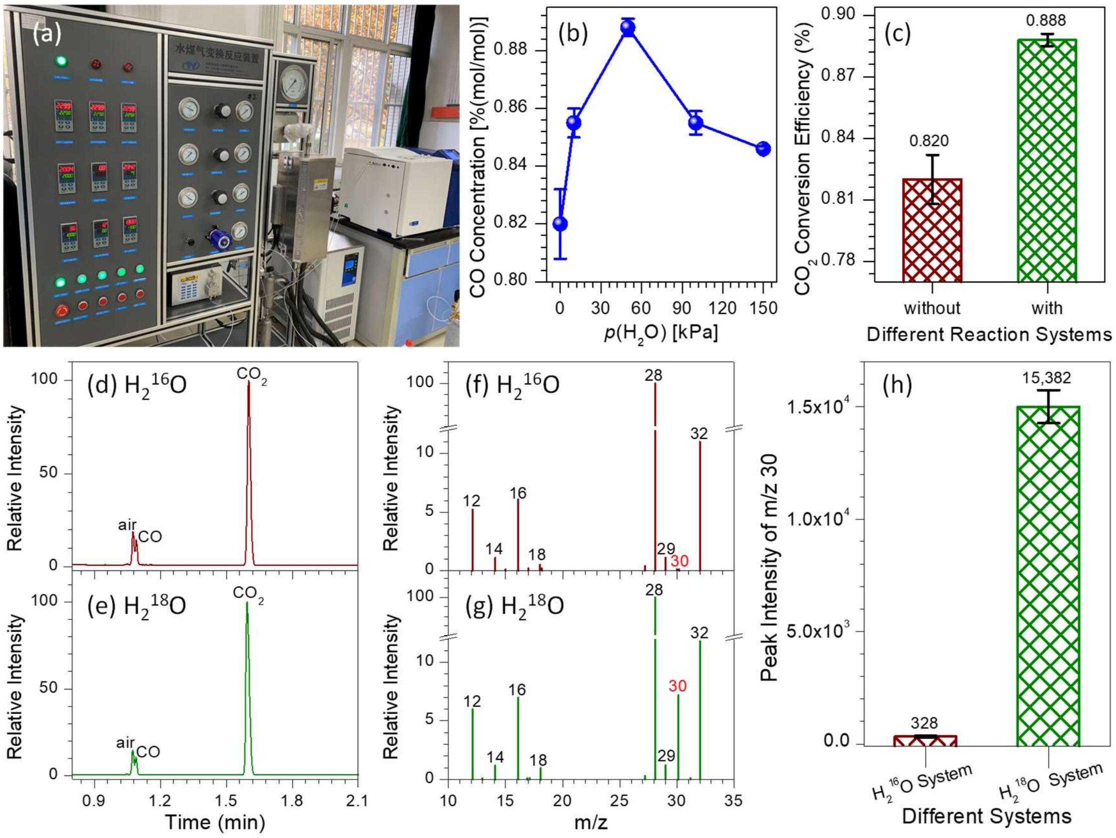

Supplementary Figure 6 | Effect of H_2O pressure on the reduction of CO_2 to CO using a water-gas shift reaction (WGSR) apparatus equipped with gas chromatography: **(a)** WGSR apparatus. **(b)** Effect of H_2O pressure on CO generation (reaction temperature: 230 ^oC; $p(CO_2)$ = 0.3 MPa, $p(H_2)$ = 2.7 MPa, and $p(H_2O)$ was controlled by the flow rate of syringe pump; n = 3). **(c)** Comparison of CO_2 conversion efficiency in the absence and presence of optimized H_2O content (50 kPa). GC chromatograms of the collected gas samples prepared in the presence of 50 kPa of **(d)** $H_2{}^{16}O$ and **(e)** $H_2{}^{18}O$. Mass spectra of CO products prepared in the presence of 50 kPa of **(f)** $H_2{}^{16}O$ and **(g)** $H_2{}^{18}O$, corresponding to CO peaks in **(d)** and **(e)**, respectively. **(h)** Comparison of the peak intensity of m/z 30 observed in **(f)** and **(g)**.

To further confirm the current conclusion in actual heterogeneous catalysis, we employed a water-gas shift reaction (WGSR) apparatus equipped with gas chromatography (**Supplementary Figure 6a**) to *online* investigate the effect of H_2O content on the reduction of CO_2 to CO, and commercial available $Cu/ZnO/Al_2O_3$ particles were used as catalyst. To well control the H_2O content, a certain amount of H_2O was continuously introduced to the reaction system by adjusting the flow rate of syringe pump. As shown in **Supplementary Figure 6b**, the regeneration amount

of CO demonstrated a first increasing trend followed by a declining one, in which it gave the optimal performance when the H_2O partial pressure in reaction system was 50 kPa. Such a pattern was in good agreement with the previous theoretical calculations by Sun *et al.*[1] Namely, H_2O could kinetically accelerate the hydrogenation on CO_2 to COOH, promoting the reverse WGSR to produce CO, whereas the too high initial partial pressure of H_2O would thermodynamically inhibit the CO_2 conversion. Although the improvement using the current reaction system was not so impressive, it indicated that the presence of H_2O could indeed promote the conversion of CO_2 to CO in heterogeneous catalysis. **Supplementary Figure 6c** compares CO_2 conversion efficiency in the absence and presence of optimized H_2O content (50 kPa). Although a pretty low conversion efficiency was observed, the results suggested that the presence of H_2O favored CO_2 conversion to CO.

For the purpose of confirming the origin of O atom in resulting CO from CO_2 reduction, we employed the above reverse WGSR to reduce CO_2 in the presence of 50 kPa of $H_2{}^{16}O$ and $H_2{}^{18}O$, respectively. Afterward, the reaction products were collected and measured *off-line* with an Agilent Technologies 7890B gas chromatograph equipped with a GS-CarbonPLOT capillary column and an Agilent Technologies 5977A mass spectrometer. **Supplementary Figures 6d** and **6e** show the corresponding chromatograms. Due to the limited separation capacity of GS-CarbonPLOT capillary column in a manual injection mode, the peaks of air and CO could not be resolved well, but both could be observed clearly. Subsequently, we identified the components of CO from the systems of $H_2{}^{16}O$ and $H_2{}^{18}O$ using mass spectrometry. As demonstrated in **Supplementary Figures 6f** and **6g**, a more abundant peak of m/z 30 appeared for the product in the presence of $H_2{}^{18}O$ than that in the presence of $H_2{}^{16}O$. According to the current reaction system, the peak of m/z 30 could be assigned to $C^{18}O$ from the interaction of CO_2 and $H_2{}^{18}O$. More importantly, after comparing the peak intensity, it is apparent that an improvement of 46.9-fold in peak intensity of m/z 30 was observed for the system of $H_2{}^{18}O$ than that in the presence of $H_2{}^{16}O$. These results not only suggest the peak m/z 30 was not from background, and also brought further solid evidence that the O atom in resulting CO originated the involved H_2O.

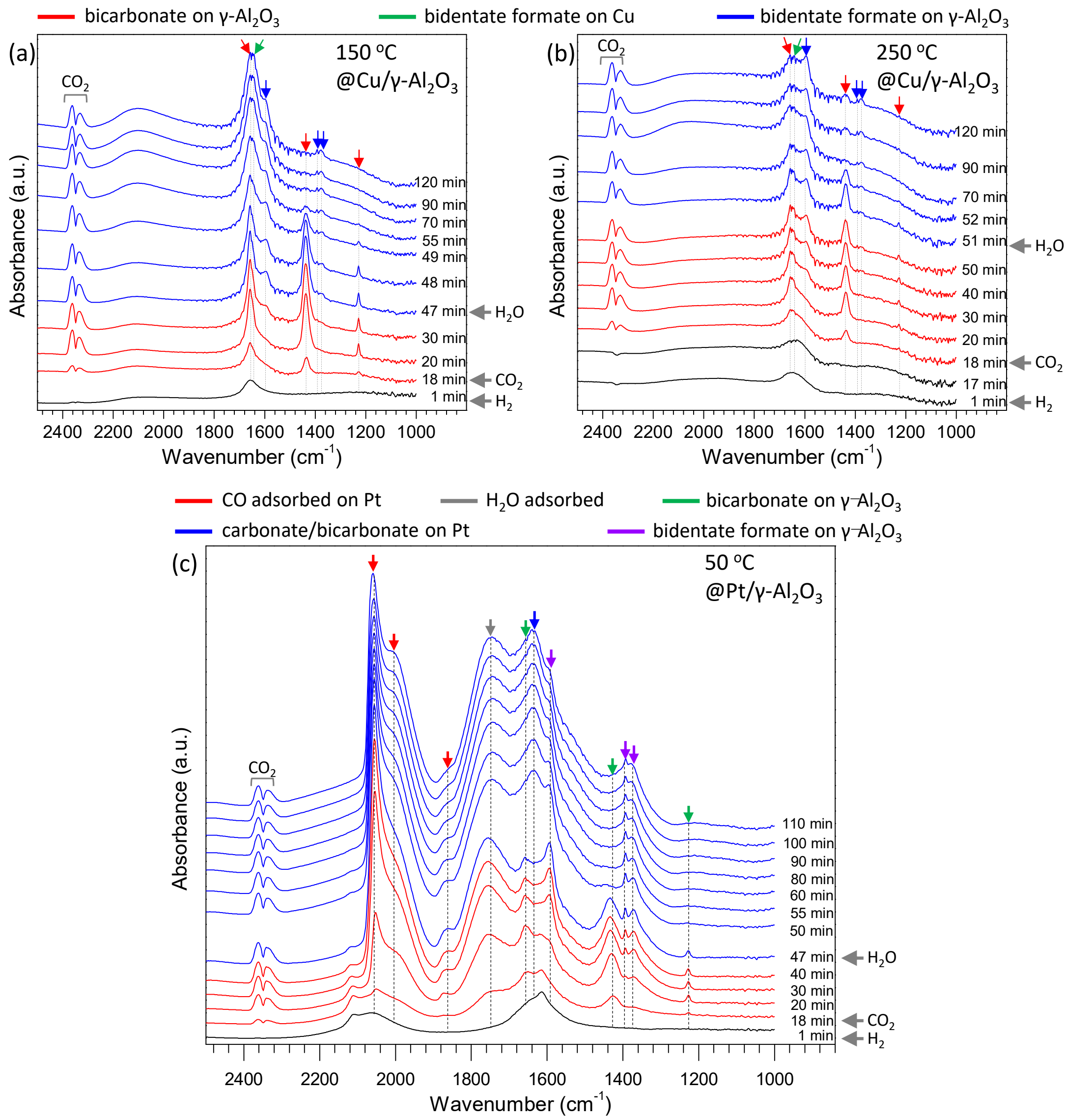

Supplementary Figure S7 | *In situ* DRIFT spectra of the resulting products on Cu/γ-Al_2O_3 or Pt/γ-Al_2O_3 with addition of H_2O into the reaction system of CO_2 and H_2 at different reaction temperatures: **(a)** 150 °C @Cu/γ-Al_2O_3, **(b)** 250 °C @Cu/γ-Al_2O_3, and **(c)** 50 °C @Pt/γ-Al_2O_3 (flow rate of 5%CO_2/95%Ar: 4 mL min^{-1}; flow rate of H_2: 3 mL min^{-1}).

In-situ DRIFTS could give more convincing results to the current study. The experiment was carried out at 150 °C and 250 °C with Cu/*γ*-Al_2O_3 as catalyst, respectively, and the corresponding results are displayed in **Supplementary Figure 7a** and **b**. It is apparent that for both reaction temperatures, the *in situ* DRIFT spectra had an analogous pattern by adding H_2O into the reaction system. Namely, after introducing CO_2 into the system (without H_2O), the absorption peak intensity of bicarbonate on *γ*-Al_2O_3 (1230 cm^{-1}, 1439 cm^{-1}, and 1670 cm^{-1}) demonstrated a gradually

increasing trend and reached a plateau. Simultaneously, the absorption peak of bidentate formate (1602 cm^{-1}) on γ-Al_2O_3 steadily emerged, but this type of absorption peak on Cu (1649 cm^{-1}) was not obvious. However, after H_2O was introduced to the reaction system, on the one hand, the absorption peak of bidentate formate on γ-Al_2O_3 (1379 cm^{-1}, 1398 cm^{-1}, and 1602 cm^{-1}) gradually increased, along with the gradual decay till disappearance of the absorption peak of bicarbonate occurred at 1230 cm^{-1} and 1439 cm^{-1}. More importantly, the absorption peak of bidentate formate on Cu (1649 cm^{-1}) exhibited a gradually increasing trend. These results indicated that H_2O played a crucial role in the generation of formate on both Cu and γ-Al_2O_3. According to our discussion on **Fig. 5a**, formate was a critical intermediate in the reduction of CO_2 to CO, which confirmed the critical role of H_2O in CO_2RR. Despite this, no obvious absorption peaks of CO adsorbed on either γ-Al_2O_3 or Cu were observed, due to its fast desorption rate or weak adsorption on both supports under the current conditions.[2]

To get more compelling results on the effect of H_2O in CO_2RR, Pt/γ-Al_2O_3 was employed as a catalyst to perform the experiments. As shown in **Supplementary Figure 7c**, after introducing H_2O into the reaction system, the absorption peak of CO adsorbed on Pt (2000 cm^{-1} and 2061 cm^{-1}) demonstrated a gradual increasing pattern with the extension of reaction period. Such a result indicated that the introduced H_2O was indeed favorable to the generation of CO in CO_2RR

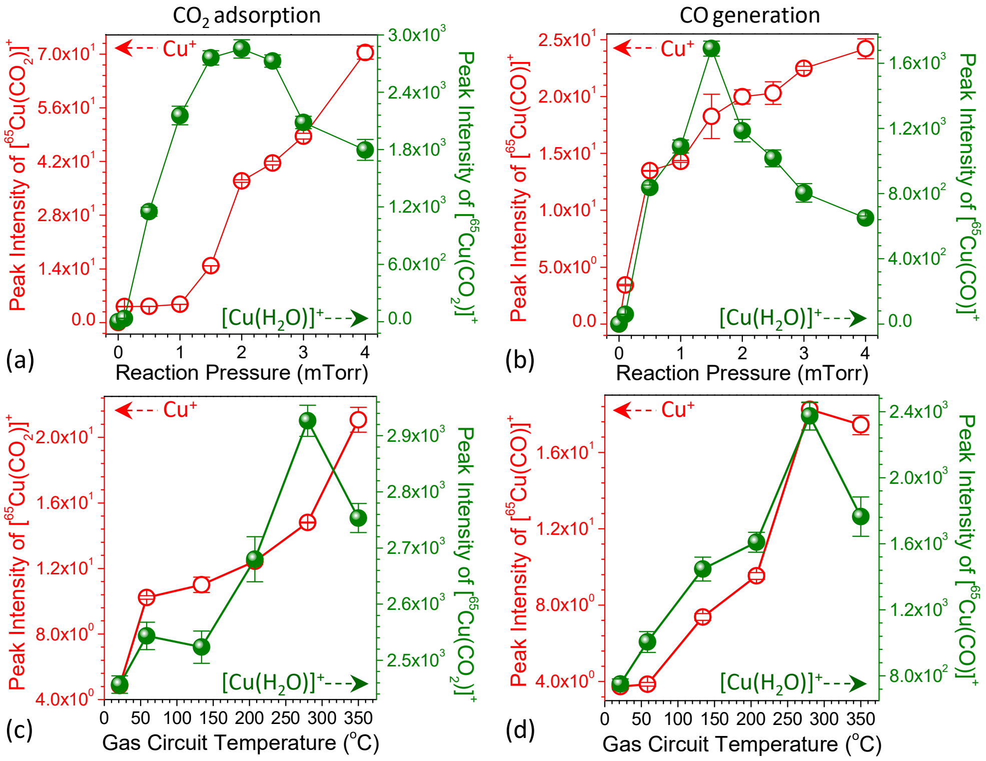

Supplementary Figure 8 | Variation of CO_2 adsorption and CO generation with reaction pressure and temperature of heating tape around the gas circuit: Effect of reaction pressure on **(a)** the adsorption CO_2 to $^{65}Cu^+$ and **(b)** CO generation by CO_2 reduction upon the catalysis of $^{65}Cu^+$ or $[^{65}Cu(H_2O)]^+$ (gas circuit temperature: 280 °C); effect of gas circuit temperature on **(c)** the adsorption CO_2 to $^{65}Cu^+$ and **(d)** CO generation by CO_2 reduction upon the catalysis of $^{65}Cu^+$ or $[^{65}Cu(H_2O)]^+$ (reaction pressure: 1.5 mTorr).

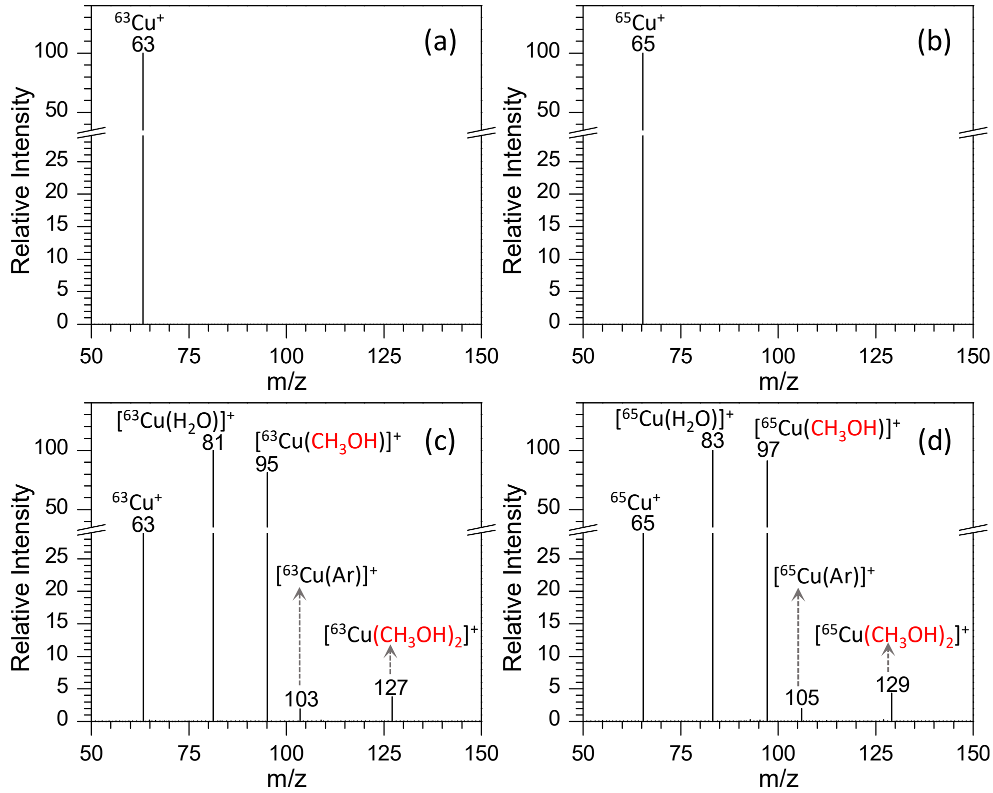

Supplementary Figure 9 | Interaction of methanol (CH_3OH, M.W. = 32) and different Cu-based species: **(a)** $^{63}Cu^+$, **(b)** $^{65}Cu^+$, **(c)** $[^{63}Cu(H_2O)]^+$, and **(d)** $[^{65}Cu(H_2O)]^+$ (gas circuit temperature: 280 °C; reaction pressure: 1.5 mTorr).

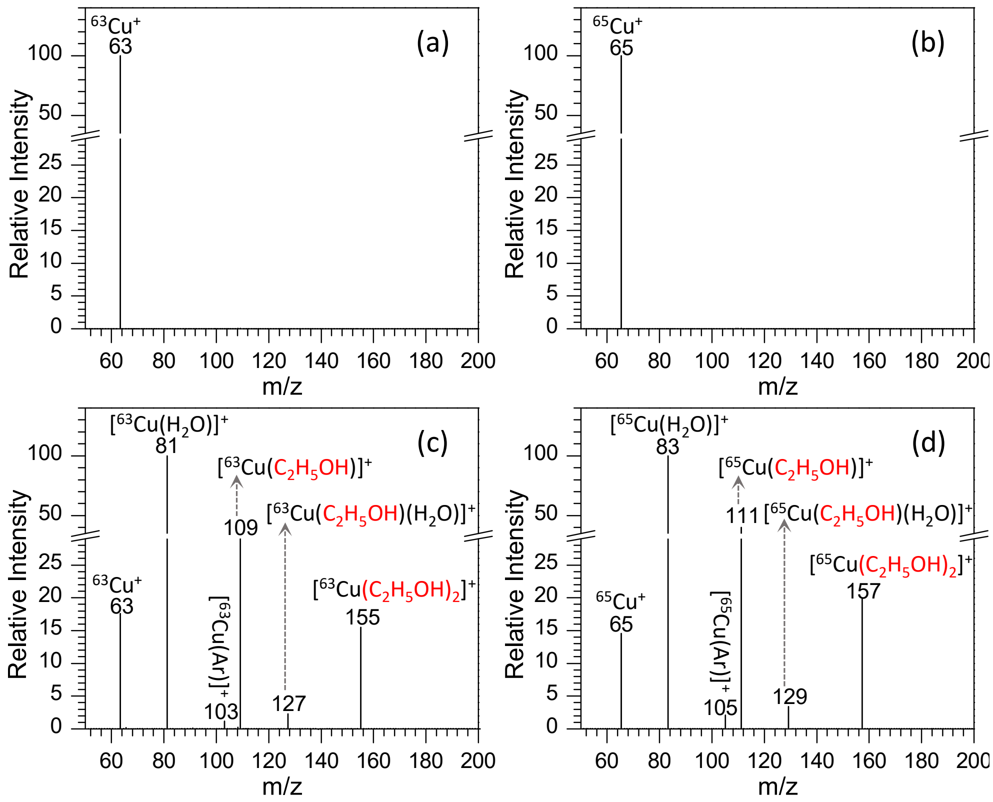

Supplementary Figure 10 | Interaction of ethanol (CH_3CH_2OH, M.W. = 46) and different Cu-based species: **(a)** $^{63}Cu^+$, **(b)** $^{65}Cu^+$, **(c)** $[^{63}Cu(H_2O)]^+$, and **(d)** $[^{65}Cu(H_2O)]^+$ (gas circuit temperature: 280 °C; reaction pressure: 1.5 mTorr).

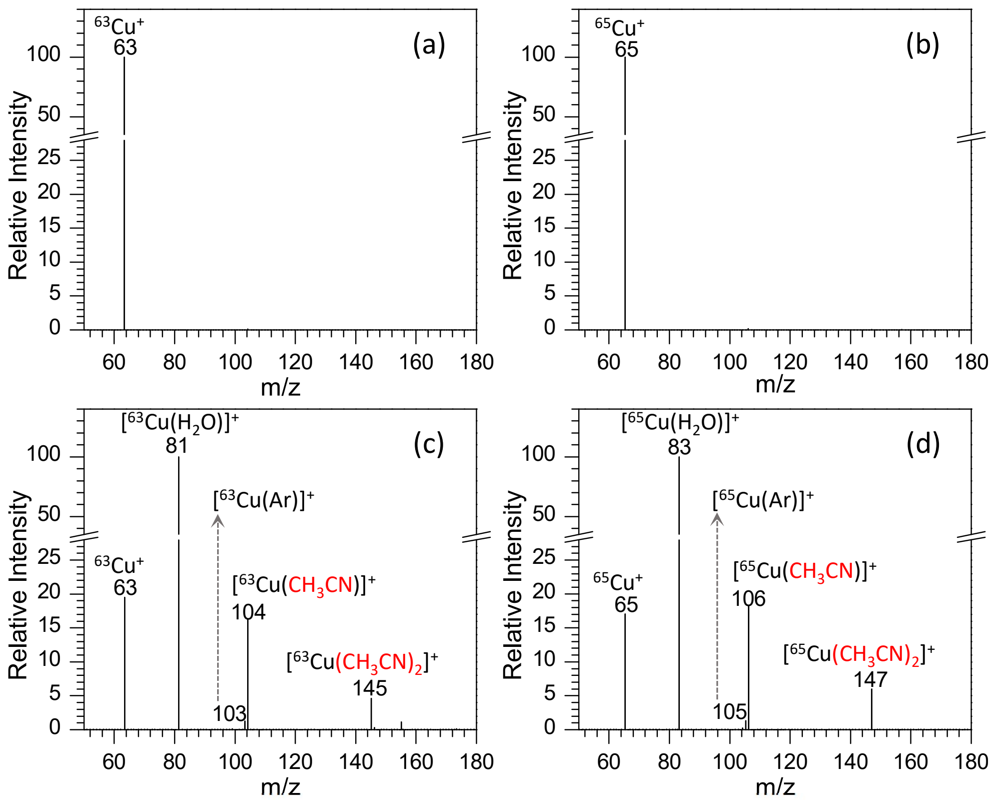

Supplementary Figure 11 | Interaction of acetonitrile (CH_3CN, M.W. = 41) and different Cu-based species: **(a)** $^{63}Cu^+$, **(b)** $^{65}Cu^+$, **(c)** $[^{63}Cu(H_2O)]^+$, and **(d)** $[^{65}Cu(H_2O)]^+$ (gas circuit temperature: 280 °C; reaction pressure: 1.5 mTorr).

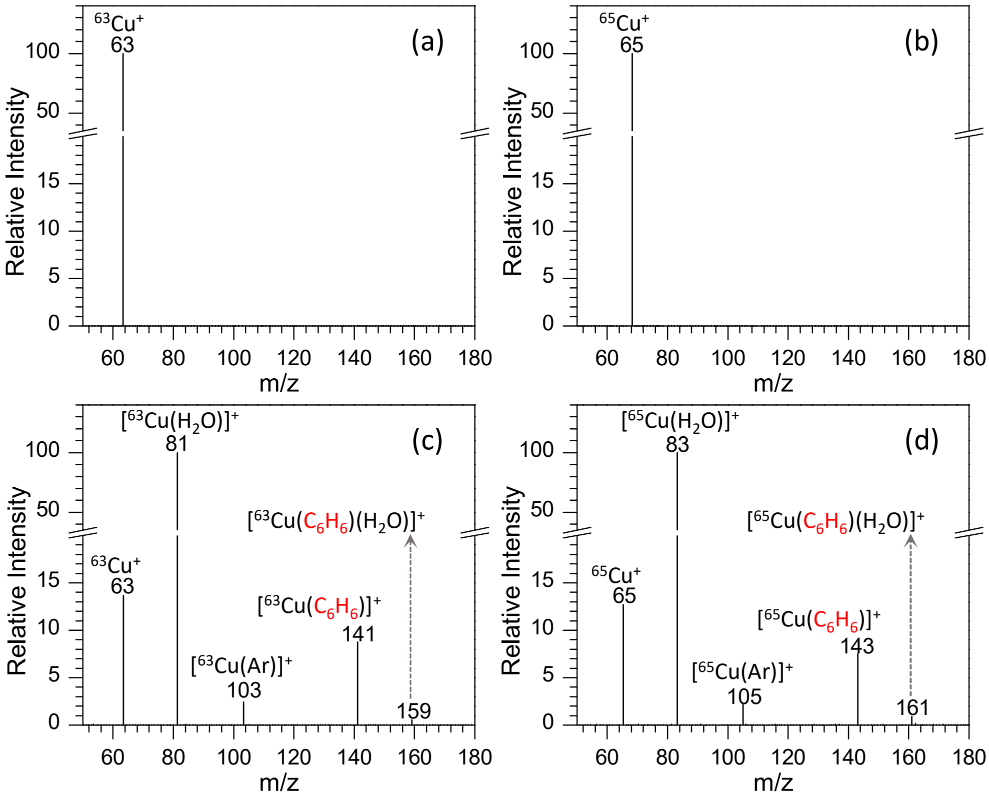

Supplementary Figure 12 | Interaction of benzene (C_6H_6, M.W. = 78) and different Cu-based species: **(a)** $^{63}Cu^+$, **(b)** $^{65}Cu^+$, **(c)** $[^{63}Cu(H_2O)]^+$, and **(d)** $[^{65}Cu(H_2O)]^+$ (gas circuit temperature: 280 °C; reaction pressure: 1.5 mTorr).

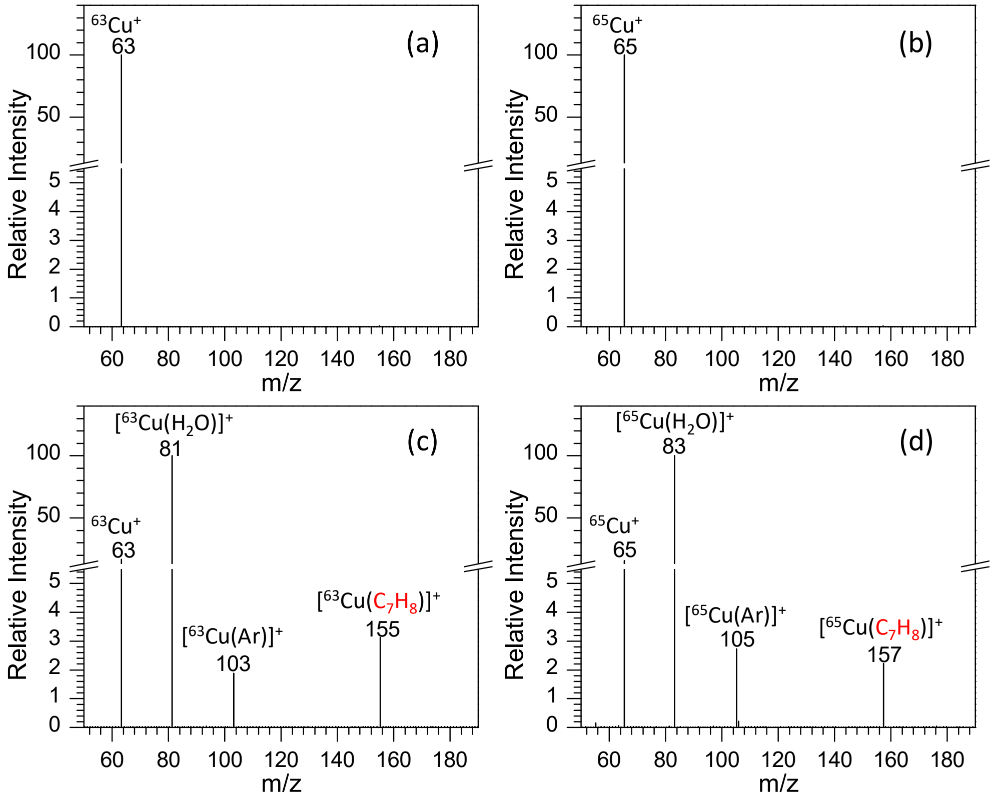

Supplementary Figure 13 | Interaction of toluene (C_7H_8, M.W. = 92) and different Cu-based species: **(a)** $^{63}Cu^+$, **(b)** $^{65}Cu^+$, **(c)** $[^{63}Cu(H_2O)]^+$, and **(d)** $[^{65}Cu(H_2O)]^+$ (gas circuit temperature: 280 ^oC; reaction pressure: 1.5 mTorr).

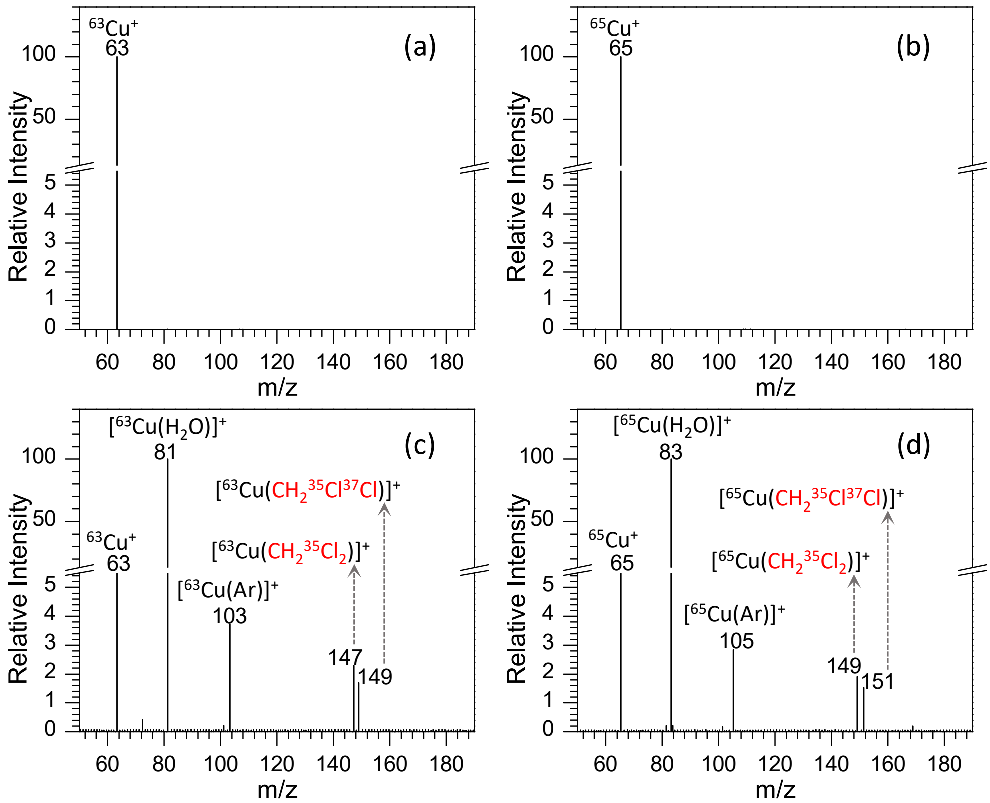

Supplementary Figure 14 | Interaction of dichloromethane ($CH_2{}^{35}Cl_2$, M.W. = 84; $CH_2{}^{35}Cl^{37}Cl$, M.W. = 86) and different Cu-based species: **(a)** $^{63}Cu^+$, **(b)** $^{65}Cu^+$, **(c)** $[^{63}Cu(H_2O)]^+$, and **(d)** $[^{65}Cu(H_2O)]^+$ (gas circuit temperature: 280 °C; reaction pressure: 1.5 mTorr).

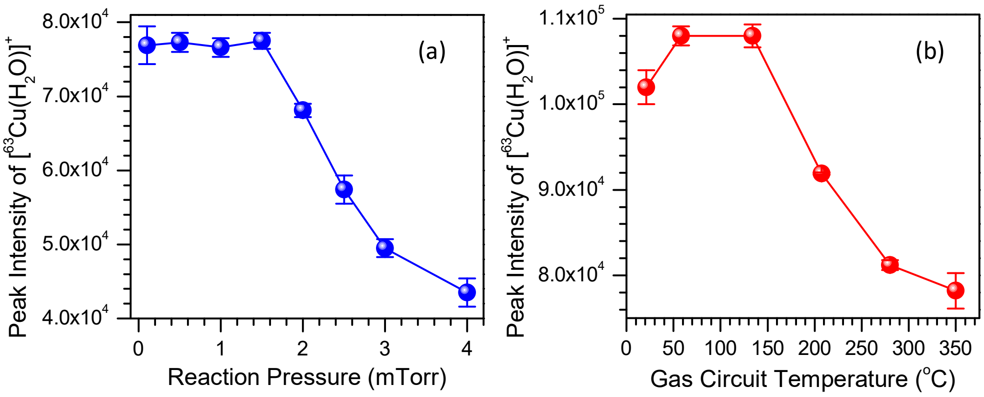

Supplementary Figure 15 | Variation in the peak intensity of $[^{63}Cu(H_2O)]^+$ with reaction pressures and gas circuit temperatures: **(a)** Effect of the reaction pressures on the peak intensity of $[^{63}Cu(H_2O)]^+$ (reaction pressure: 1.5 mTorr; *n* = 5); **(b)** Effect of the gas circuit temperatures on the peak intensity of $[^{\mathbf{63}}Cu(H_2O)]^+$ (reaction temperature: 280 °C; n = 5).

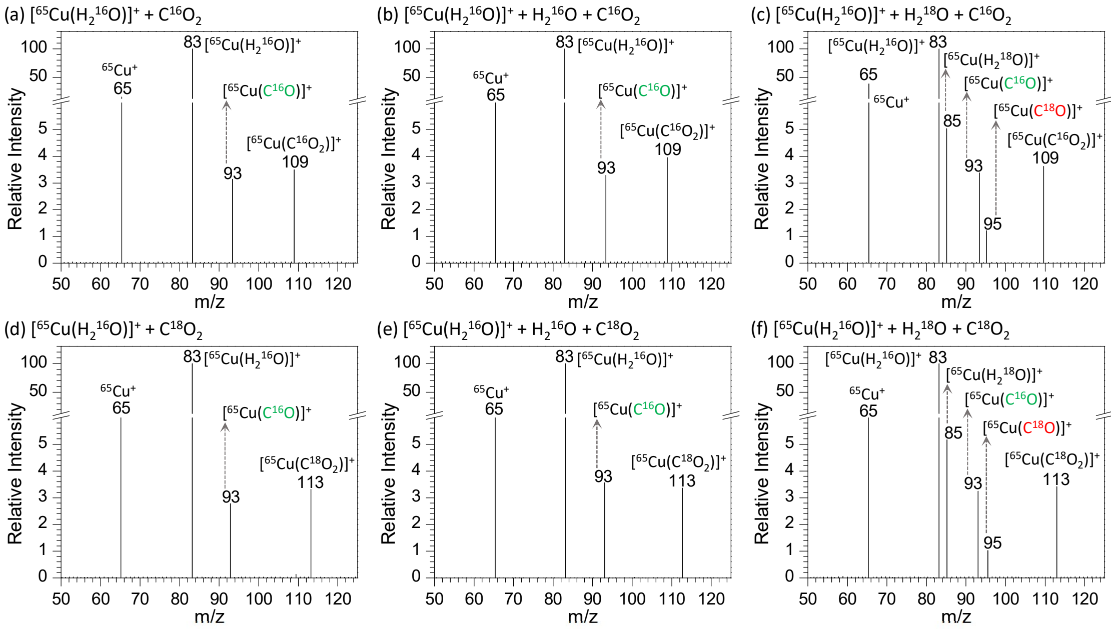

Supplementary Figure 16 | Isotope-labeling MS measurement results under different ^{65}Cu-based reaction systems: **(a)** $[^{65}Cu(H_2{}^{16}O)]^+ + C^{16}O_2$, **(b)** $[^{65}Cu(H_2{}^{16}O)]^+ + H_2{}^{16}O + C^{16}O_2$, **(c)** $[^{65}Cu(H_2{}^{16}O)]^+ + H_2{}^{18}O + C^{16}O_2$, **(d)** $[^{65}Cu(H_2{}^{16}O)]^+ + C^{18}O_2$, **(e)** $[^{65}Cu(H_2{}^{16}O)]^+ + H_2{}^{16}O + C^{18}O_2$, and **(f)** $[^{65}Cu(H_2{}^{16}O)]^+ + H_2{}^{18}O + C^{18}O_2$ (gas circuit temperature: 280 °C; reaction pressure: 1.5 mTorr).

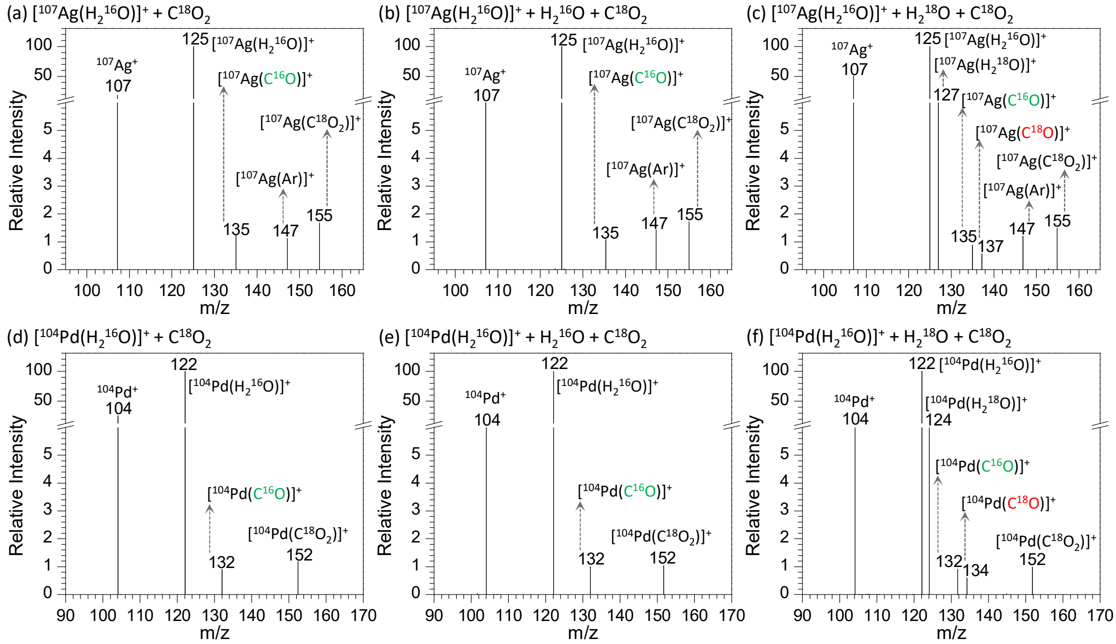

Supplementary Figure 17 | Isotope-labeling MS measurement results under different ^{107}Ag/^{104}Pd-based reaction systems: **(a)** $[^{107}Ag(H_2{}^{16}O)]^+$ + $C^{18}O_2$, **(b)** $[^{107}Ag(H_2{}^{16}O)]^+$ + $H_2{}^{16}O$ + $C^{18}O_2$, **(c)** $[^{107}Ag(H_2{}^{16}O)]^+$ + $H_2{}^{18}O$ + $C^{18}O_2$, **(d)** $[^{104}Pd(H_2{}^{16}O)]^+$ + $C^{18}O_2$, **(e)** $[^{104}Pd(H_2{}^{16}O)]^+$ + $H_2{}^{16}O$ + $C^{18}O_2$, and **(f)** $[^{104}Pd(H_2{}^{16}O)]^+$ + $H_2{}^{18}O$ + $C^{18}O_2$ (gas circuit temperature: 280 ^{o}C; reaction pressure: 1.5 mTorr).

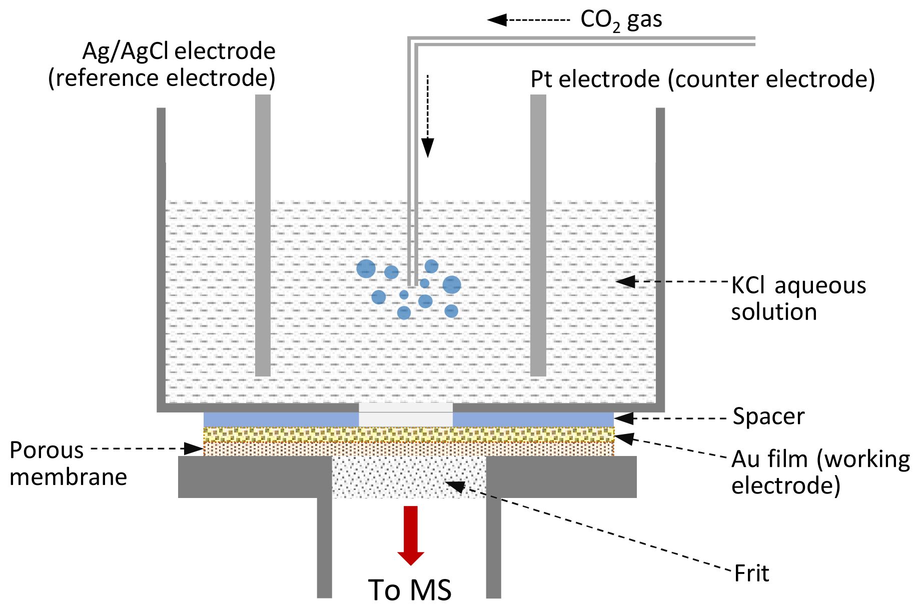

Supplementary Figure 18 | Differential electrochemical mass spectrometer for the electrochemical reduction of CO_2 to CO and *in situ* mass spectrometer monitoring of resulting reaction products.

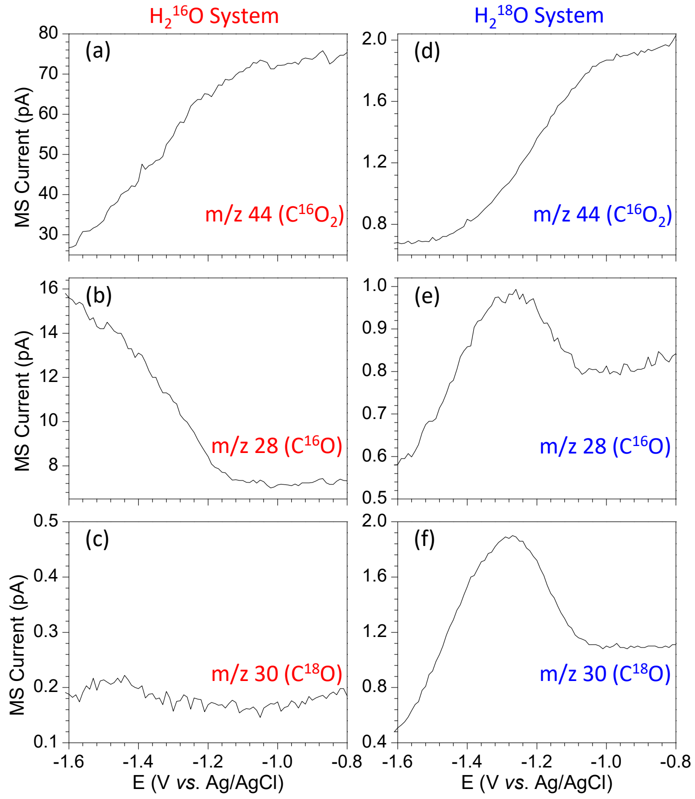

Supplementary Figure 19 | Simultaneous measurement of the MS current of different ions using an *in situ* differential electrochemical mass spectrometer (Au electrode): **(a)** m/z 44, **(b)** m/z 28, and **(c)** m/z 30 in the system of $H_2{}^{16}O$, and **(d)** m/z 44, **(e)** m/z 28, and **(f)** m/z 30 in the system of $H_2{}^{18}O$ for Au electrode in 0.5 M of KCl aqueous solution.

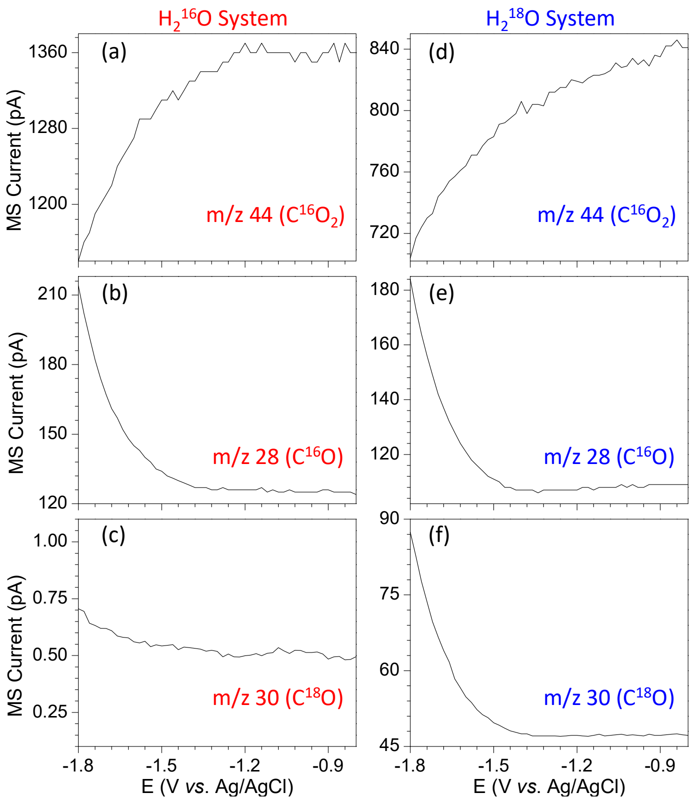

Supplementary Figure 20 | Simultaneous measurement of the MS current of different ions using an *in situ* differential electrochemical mass spectrometer (Ag electrode): **(a)** m/z 44, **(b)** m/z 28, and **(c)** m/z 30 in the system of $H_2{}^{16}O$, and **(d)** m/z 44, **(e)** m/z 28, and **(f)** m/z 30 in the system of $H_2{}^{18}O$ for Ag electrode in 0.5 M of $KHCO_3$ aqueous solution.

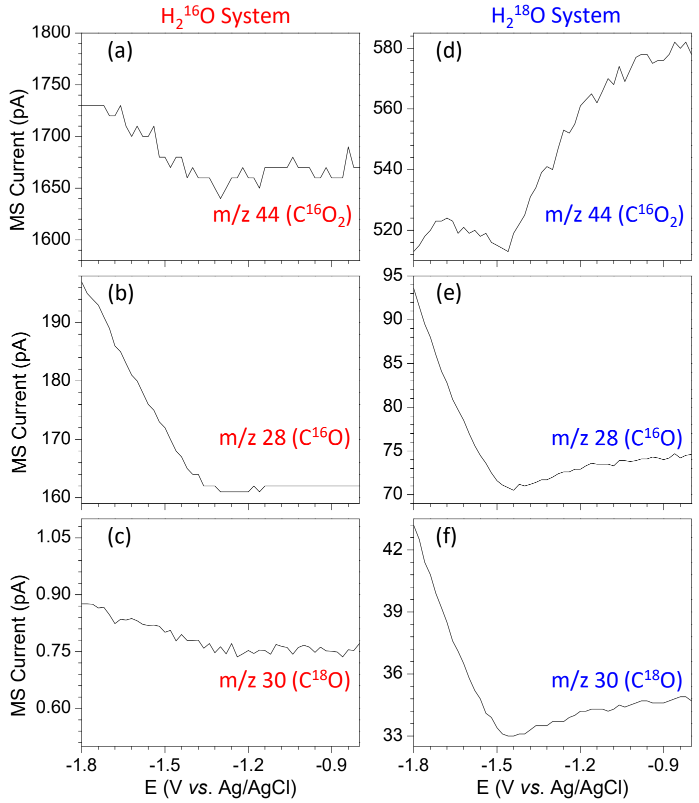

Supplementary Figure 21 | Simultaneous measurement of the MS current of different ions using an *in situ* differential electrochemical mass spectrometer (Pd electrode): **(a)** m/z 44, **(b)** m/z 28, and **(c)** m/z 30 in the system of $H_2{}^{16}O$, and **(d)** m/z 44, **(e)** m/z 28, and **(f)** m/z 30 in the system of $H_2{}^{18}O$ for Pd electrode in 0.5 M of $KHCO_3$ aqueous solution.

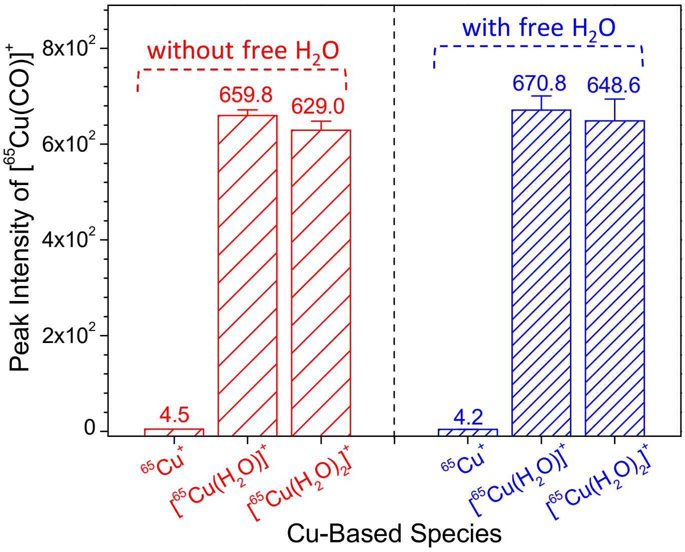

Supplementary Figure 22 | Effects of coordinated H_2O number ($^{65}Cu^+$, $[^{65}Cu(H_2O)]^+$ and $[^{65}Cu(H_2O)_2]^+$) and free H_2O on the reduction of CO_2 to CO under different Cu-based catalytic systems (The free H_2O was injected into gas circuit system by an injector; gas circuit temperature: 280 ^oC; reaction pressure: 1.5 mTorr; n = 5).

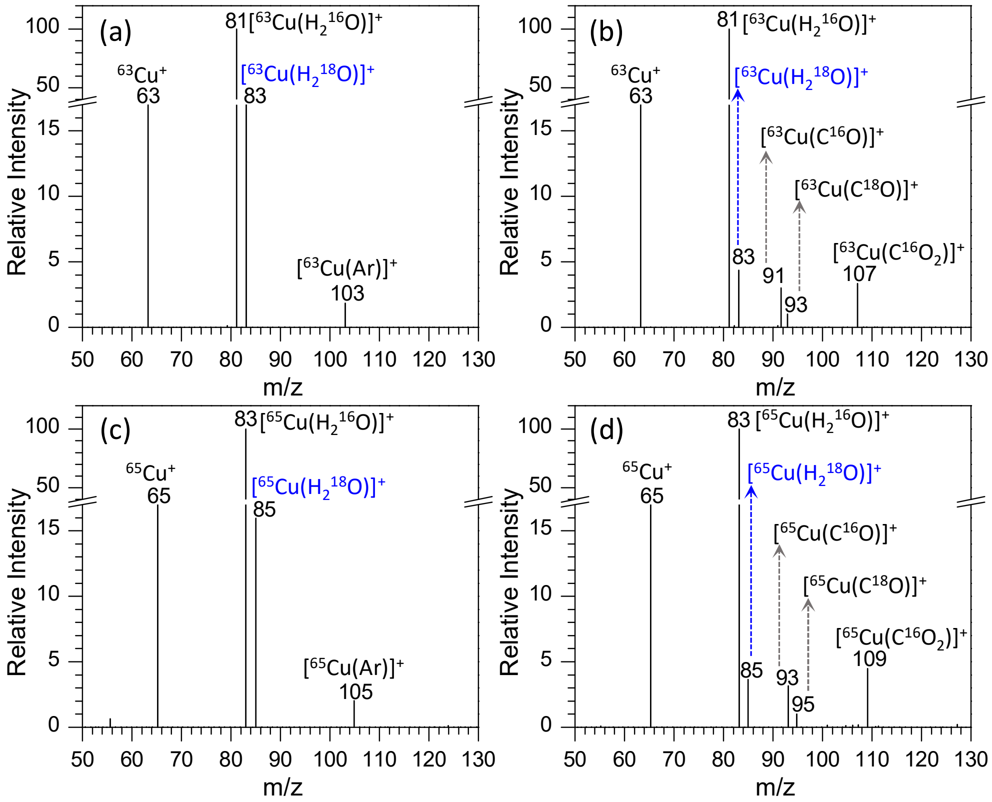

Supplementary Figure 23 | Mass spectra of the different systems after interaction between free $H_2{}^{18}O$ and CO_2: **(a)** $[^{63}Cu(H_2{}^{16}O)]^+$ + $H_2{}^{18}O$, **(b)** $[^{63}Cu(H_2{}^{16}O)]^+$ + $H_2{}^{18}O$ + $C^{16}O_2$, **(c)** $[^{65}Cu(H_2{}^{16}O)]^+$ + $H_2{}^{18}O$, and **(d)** $[^{65}Cu(H_2{}^{16}O)]^+$ + $H_2{}^{18}O$ + $C^{16}O_2$ (gas circuit temperature: 280 °C; reaction pressure: 1.5 mTorr).

Note: $H_2{}^{18}O$ was injected into the $^{16}CO_2$ gas circuit system by an injector; $[^{63}Cu(Ar)]^+$ and $[^{65}Cu(Ar)]^+$ were due to the reaction between $[^{63/65}Cu(H_2O)]^+$ or $^{63/65}Cu^+$ with argon the as collision gas.

From **Supplementary Figure 23a**, it is clear that when only pure argon was involved in the gas circuit system, intensive peak at m/z 83 (blue, $[^{63}Cu(H_2{}^{18}O)]^+$) was observed due to the interaction of $H_2{}^{18}O$ and dissociated $^{63}Cu^+$ or $[^{63}Cu(H_2{}^{16}O)]^+$. When 5% of CO_2 was involved in the mixed gas (95:5 argon/CO_2), the relative intensity of $[^{63}Cu(H_2{}^{18}O)]^+$ (blue, m/z 83) sharply decreased (**Supplementary Figure 23b**), indicating that the reaction between $^{16}CO_2$ and injected free $H_2{}^{18}O$. A same case was observed for the ^{65}Cu-based system (**Supplementary Figure 23c, d**).

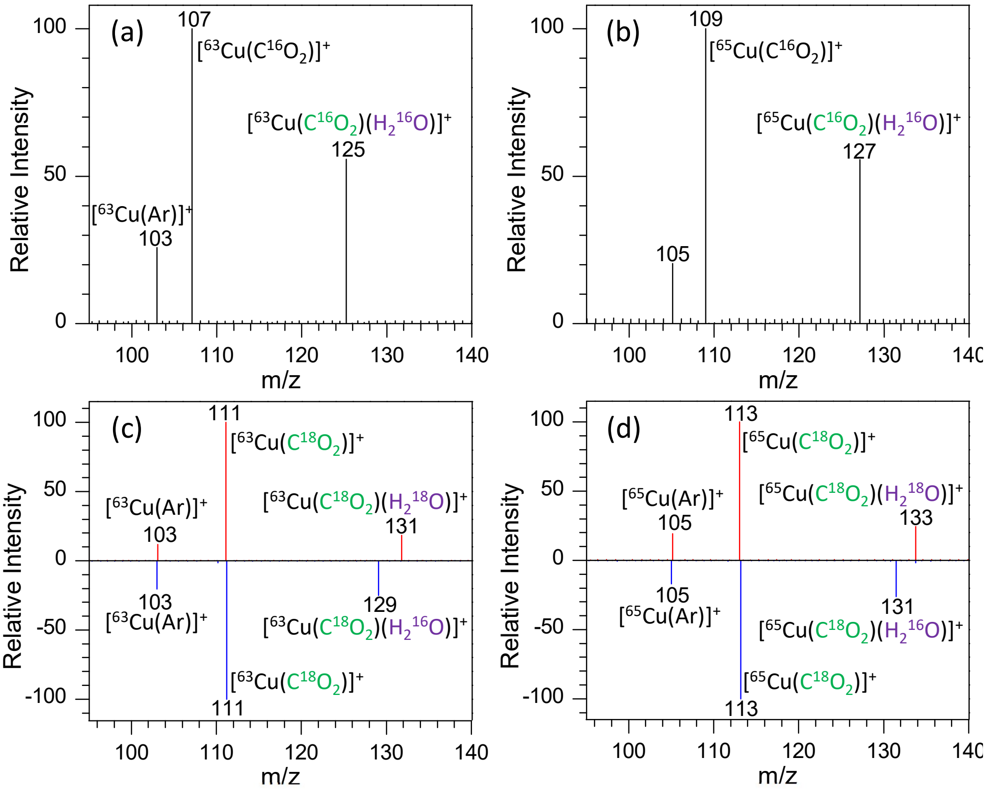

Supplementary Figure 24 | Mass spectra of the different reaction systems for capturing $[Cu(CO_2)(H_2O)]^+$ intermediates: **(a)** $[^{63}Cu(H_2{}^{16}O)]^+$ + $C^{16}O_2$ + $H_2{}^{16}O$, **(b)** $[^{65}Cu(H_2{}^{16}O)]^+$ + $C^{16}O_2$ + $H_2{}^{16}O$, **(c)** $[^{63}Cu(H_2{}^{16}O)]^+$ + $C^{18}O_2$ + $H_2{}^{18}O$, **(d)** $[^{65}Cu(H_2{}^{16}O)]^+$ + $C^{18}O_2$ + $H_2{}^{18}O$ (gas circuit temperature: 280 °C; reaction pressure: 1.5 mTorr).

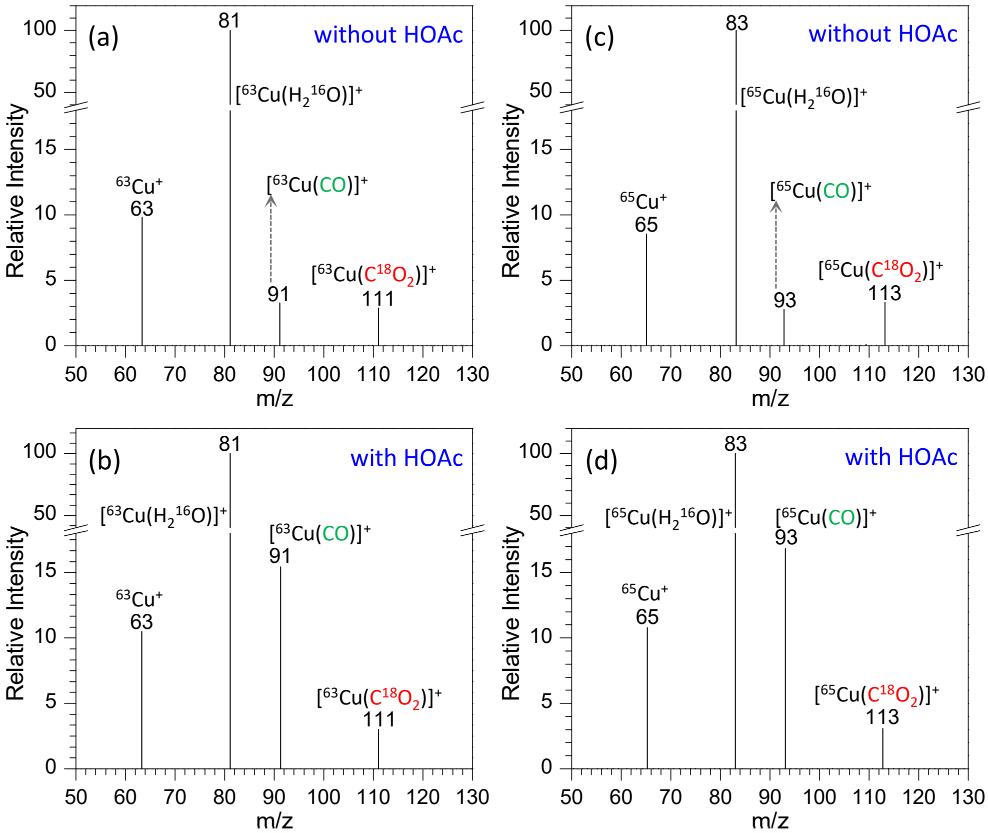

Supplementary Figure 25 | Mass spectra of the different reaction systems without and with HOAc: **(a)** $[^{63}Cu(H_2{}^{16}O)]^+$ + $C^{18}O_2$ without HOAc (acetic acid), **(b)** $[^{63}Cu(H_2{}^{16}O)]^+$ + $C^{18}O_2$ with HOAc, : **(c)** $[^{65}Cu(H_2{}^{16}O)]^+$ + $C^{18}O_2$ without HOAc (acetic acid), and **(d)** $[^{65}Cu(H_2{}^{16}O)]^+$ + $C^{18}O_2$ with HOAc (gas circuit temperature: 280 °C; reaction pressure: 1.5 mTorr).

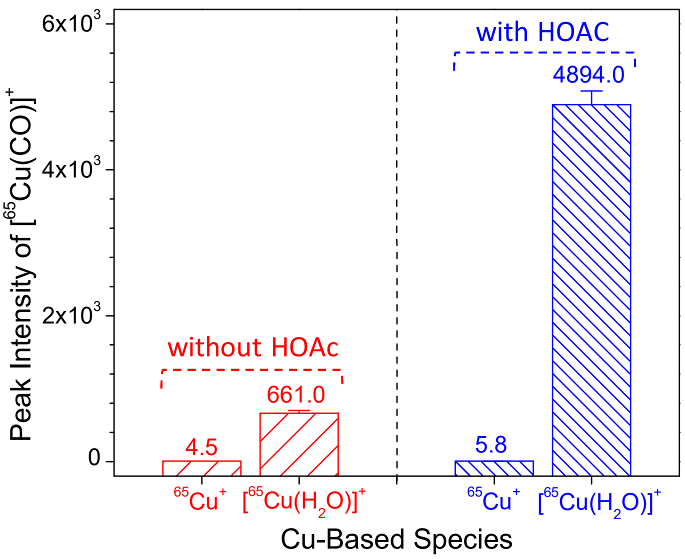

Supplementary Figure 26 | Influence of the extraneous acid on the generation of CO (The used acidic acid, HOAc, was injected into gas circuit system by an injector; gas circuit temperature: 280 °C; reaction pressure: 1.5 mTorr; n = 5).

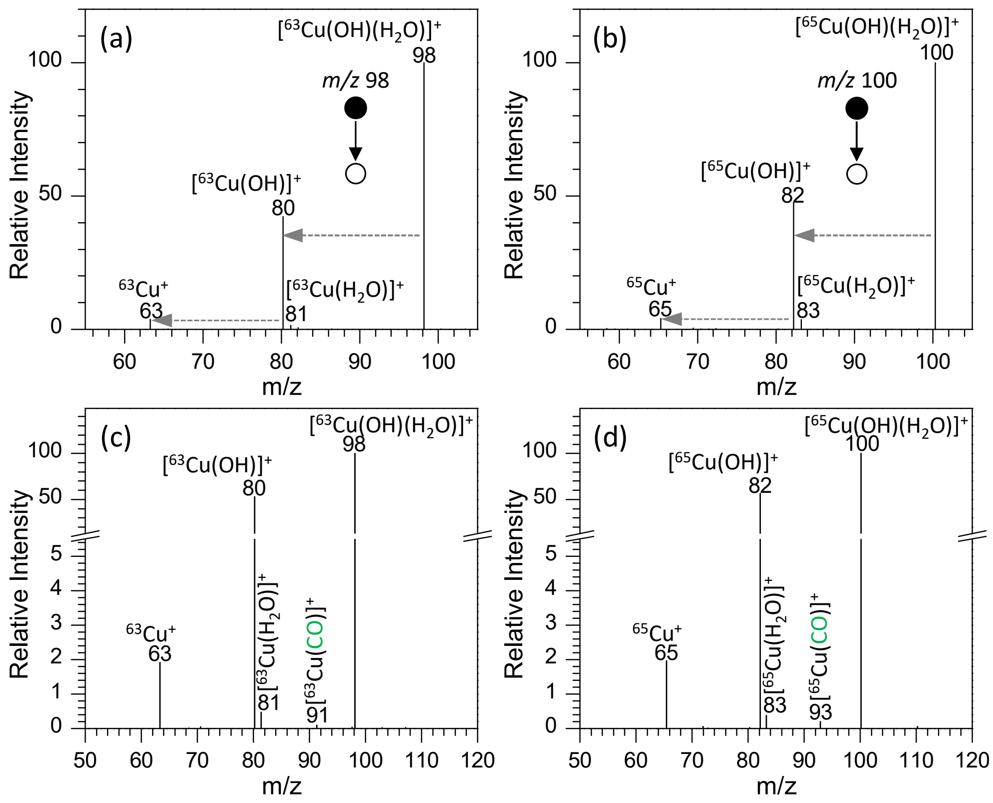

Supplementary Figure 27 | MS/MS spectra of $[^{63}Cu(OH)(H_2O)]^+$ and $[^{65}Cu(OH)(H_2O)]^+$ and their catalysis effects to CO_2 reduction: MS/MS spectra of **(a)** m/z 98 and **(b)** m/z 100; mass spectra of reduction of CO_2 to CO under two different Cu-based catalytic systems: **(c)** $[^{63}Cu(OH)(H_2O)]^+$ and **(d)** $[^{65}Cu(OH)(H_2O)]^+$ (gas circuit temperature: 280 ^oC; reaction pressure: 1.5 mTorr).

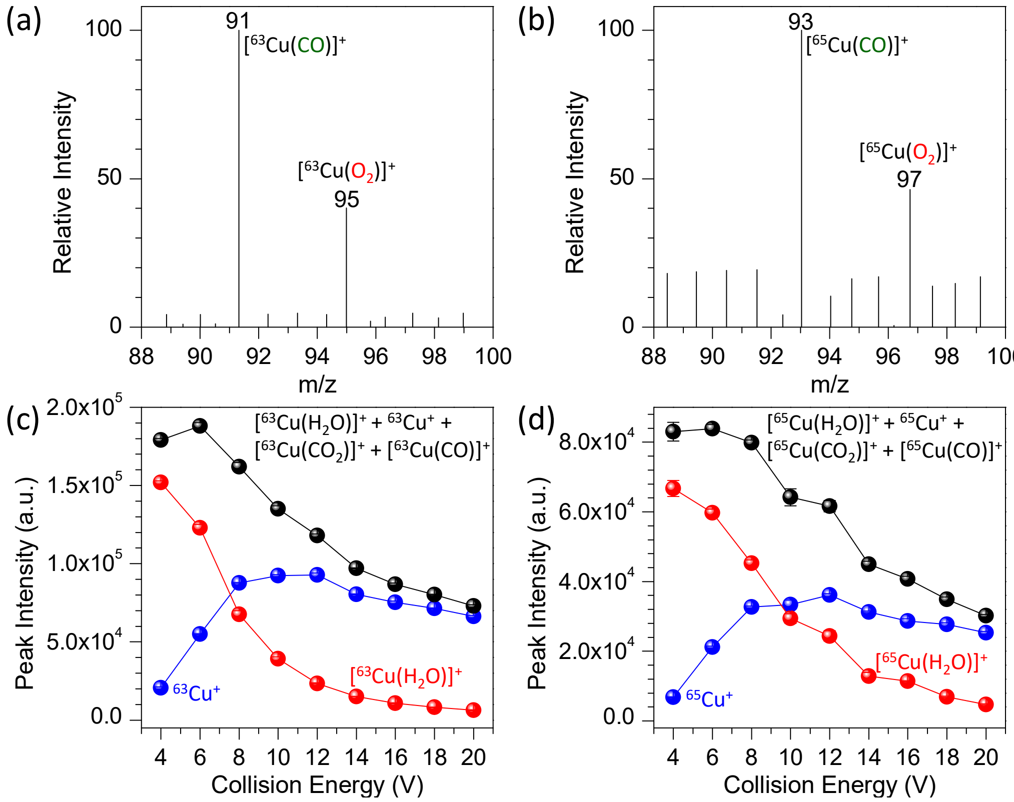

Supplementary Figure 28 | Mass spectra of the products by interaction between $[Cu(H_2O)]^+$ and CO_2 and variation in the peak intensity of different Cu-based species with increasing collision energy: Mass spectra of the products by interaction **(a)** between $[^{63}Cu(H_2O)]^+$ and CO_2 and **(b)** between $[^{65}Cu(H_2O)]^+$ and CO_2; Variation in the peak intensity of different **(c)** ^{63}Cu-based and **(d)** ^{65}Cu-based species with increasing collision energy (gas circuit temperature: 280 °C; reaction pressure: 1.5 mTorr).

As is well-known, the Q2 region of commercial TSQ mass spectrometer (**Fig. 1**) is generally employed for collision-induced dissociation (CID) of gas phase ions, in which the selected ions are accelerated by applying an electrical potential (5 V of AC voltage in this work) to increase the ion kinetic energy and then allowed to collide with neutral molecules (e.g., argon in this work). In the collision, some of the kinetic energy is converted into internal energy which results in bond breakage and the fragmentation of the molecular ion into smaller fragments (https://en.wikipedia.org/wiki/collision-induced dissociation). In the current investigation, it involves the reactants of $[Cu(H_2O)]^+$, Cu^+, H_2O, and CO_2 in the Q2 region during the reaction/collision between $[Cu(H_2O)]^+$ and CO_2. Along with them, $[Cu(H_2CO_3)]^+$ or H_2CO_3 would be generated upon the collision between $[Cu(H_2O)]^+$/H_2O and CO_2 (**Supplementary Figure 24**). Subsequently, on the

one hand, H_2CO_3 dissociates through Eqs. (1) and (2), and the resulting H^+ ions would supply the necessary protons in **Fig. 5a** in the main text. On the other hand, many prior studies[3-7] have indicated that electrons could be dissociated from anions [e.g., $PtBr_6^{2-}$, $Pd(CN)_4^{2-}$, $Ru^3Co(CO)^{13-}$, CO_3^-, and CO_2^-] in the CID of mass spectrometry. Based on the above fact, it is speculated that the required electrons in this study could be generated following Eqs. (3)-(5) in the dissociation of CO_3^{2-}.

$$H_2CO_3 \rightarrow H^+ + HCO_3^- \quad (1)$$

$$HCO_3^- \rightarrow H^+ + CO_3^{2-} \quad (2)$$

$$CO_3^{2-} \rightarrow CO_3^- + e^- \quad (3)$$

$$CO_3^- \rightarrow CO_2 + O^- \quad (4)$$

$$O^- + O^- \rightarrow O_2 + 2e^- \quad (5)$$

If the above assumption was correct, O_2 should be produced. After examining the mass spectrum from the reaction of $[Cu(H_2O)]^+$ and CO_2, $[Cu(O_2)]^+$ ions, namely m/z 95 for $[^{63}Cu(O_2)]^+$ and 97 for $[^{65}Cu(O_2)]^+$, were indeed captured as shown in **Supplementary Figure 28a** and **b**. This fact suggested that in CO_2RR, the dissociation of H_2CO_3 was likely to be one of the electron sources.

In addition, we also explored the possibility of generating necessary electrons from Cu-based species in the CID, namely Eq. (6) or (7). If this route was feasible, a higher collision energy in CID would favor a more amount of electrons, thereby leading to a decrease in the total amount of Cu(I)-based species (e.g., $[Cu(H_2O)]^+$, Cu^+, $[Cu(CO)]^+$, and $[Cu(CO_2)]^+$). Otherwise, the amount of Cu-based species would keep constant.

$$Cu^+ \rightarrow Cu^{2+} + e^- \quad (6)$$

$$[Cu(H_2O)]^+ \rightarrow [Cu(H_2O)]^{2+} + e^- \quad (7)$$

To confirm the above possibility, in the reaction between $[Cu(H_2O)]^+$ and CO_2 we enhanced the collision energy ranging from 4 to 20 V by maintaining other parameters constant. With the increase in the collision energy, the dissociation possibility of Cu^+ to Cu^{2+} or $[Cu(H_2O)]^+$ to $[Cu(H_2O)]^{2+}$ would increase. To evaluate the total amount of Cu-based species, we summed the peak intensity of $[Cu(H_2O)]^+$, Cu^+, $[Cu(CO)]^+$, and $[Cu(CO_2)]^+$ collected from the corresponding mass spectra. As shown in **Supplementary Figure 28c** and **d**, the amount of Cu(I)-based species

presented a deceasing trend with increasing the collision energy, indicating that in the reaction of $[Cu(H_2O)]^+$ and CO_2, Cu(I)-based species would become others such as Cu(II)-based species via Eqs. (6) and (7), metallic Cu by reacting with the generated electrons from H_2CO_3 or transferring their charges to Ar gas by generation of Ar^+ (m/z 40). However, no direct evidence was gained to confirm the generation of Cu(II)-based species and Ar^+ using the current technique. In our opinion, the reduction of Cu(I)-based species to metallic Cu was highly possible because the required electrons were available in the current system, as well as the documented references.[8,9]

From the above discussion, it is apparent that there is at least more than one route to generate necessary electrons for supplying CO_2RR in the current study. Although there was no H_2 introduction or generation involved in the current work, the electrons offered opportunity to reduce CO_2 to CO.

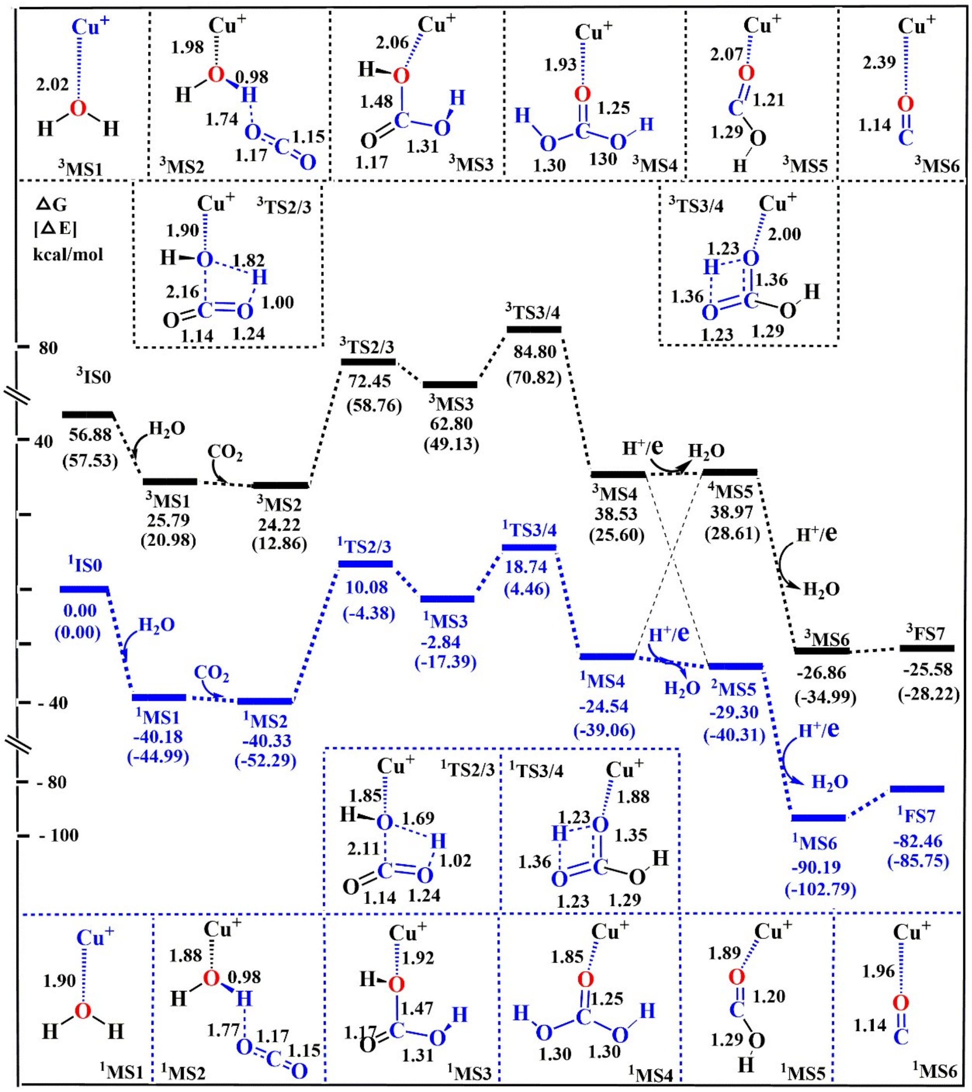

Supplementary Figure 29 | Reaction pathways of CO_2 reduction to CO catalyzed by Cu^+ and H_2O. The structural diagrams with selected geometric parameters of the IS, TS and FS for every reaction step are shown in the inset.

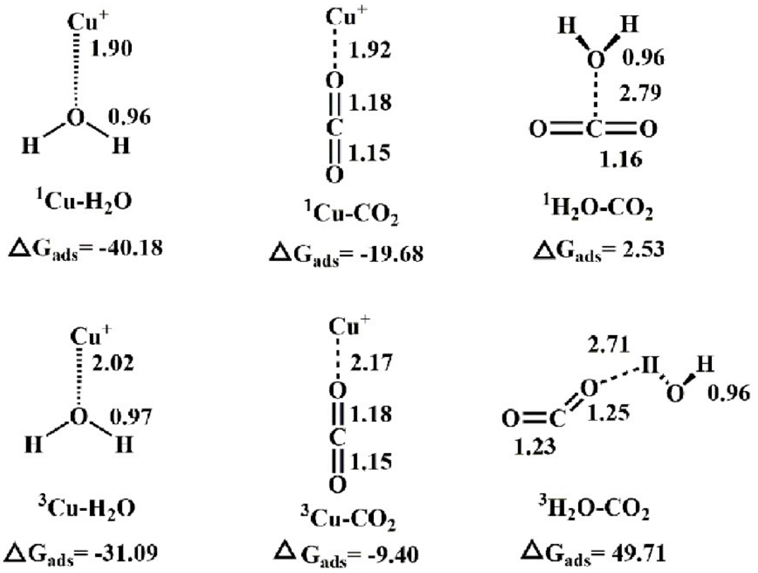

Supplementary Figure 30 | Optimal geometric structures and interaction energies among $^1CO_2$, $^1Cu^+$ and ^1H_2O at the B2PLYP/cc-PVTZ/Aug-cc-pVTZ-PP level. Bond lengths are in angstroms and energies in Kcal/mol.

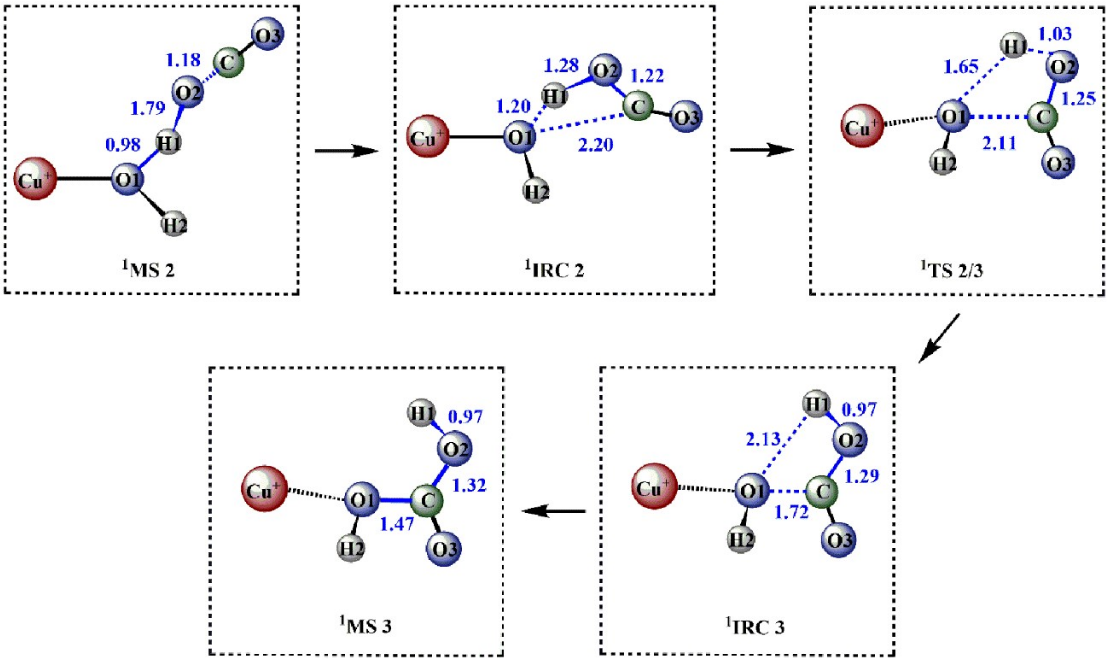

Supplementary Figure 31 | Schematic diagram of the bond breaking/making in the single step along IRC calculations of the ^{1}TS2/3 at the B2PLYP/cc-PVTZ/Aug-cc-pVTZ-PP level.

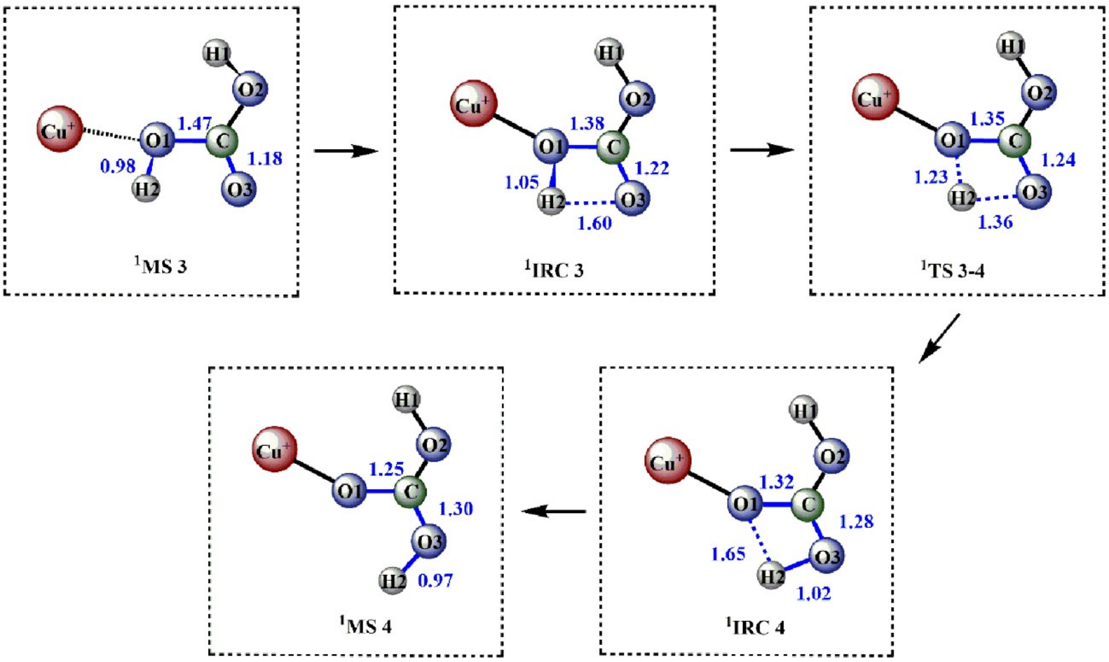

Supplementary Figure 32 | Schematic diagram of the bond breaking/making in the single step along IRC calculations of the ^{1}TS3/4 at the B2PLYP/cc-PVTZ/Aug-cc-pVTZ-PP level.

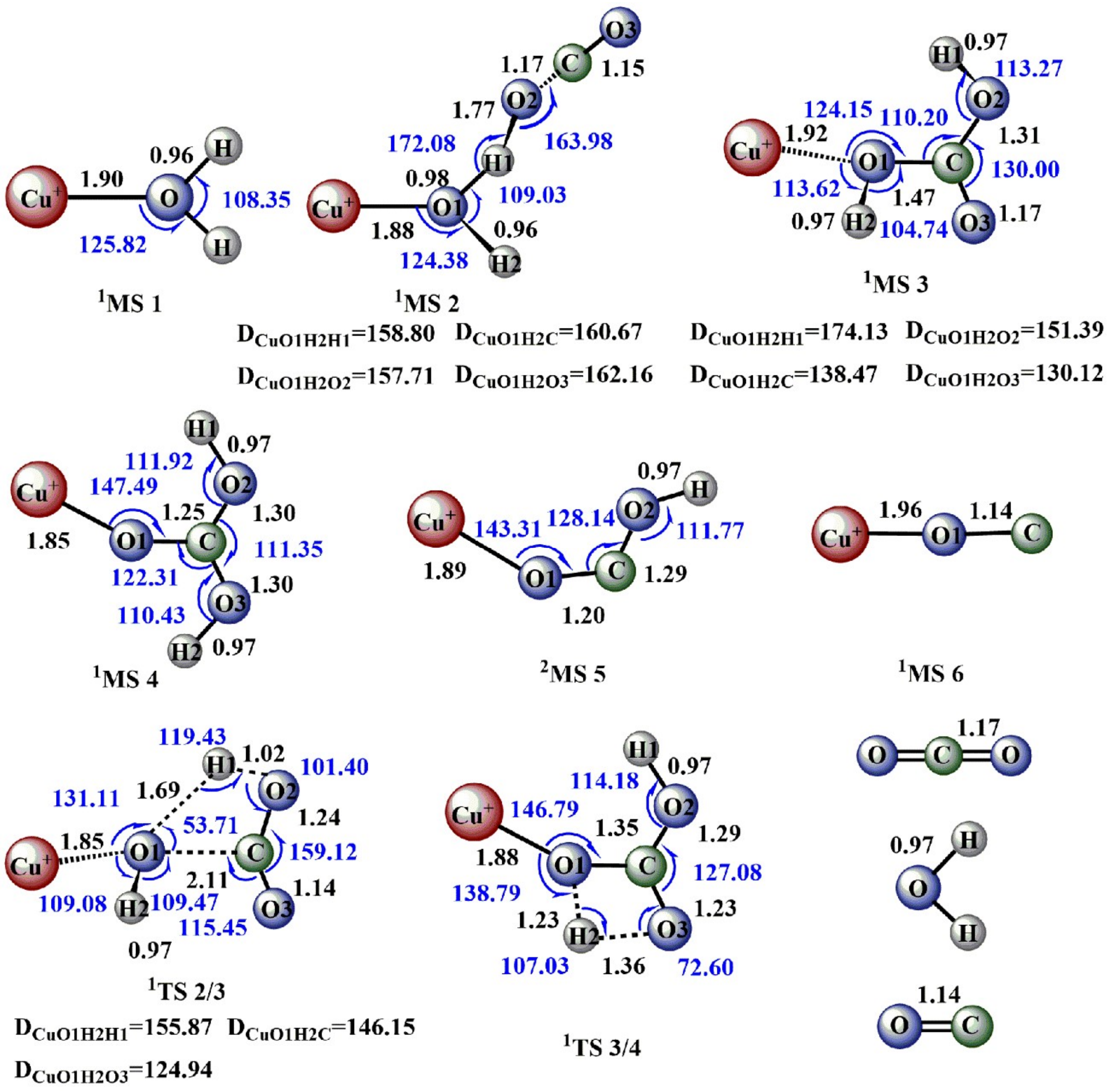

Supplementary Figure 33 | Optimal geometric structure of singlet intermediates and transition states of the reduction of CO_2 to CO catalyzed by on Cu^+ and H_2O at the B2PLYP/cc-PVTZ/Aug-cc-pVTZ-PP level. Bond lengths are in angstroms and angles in degrees.

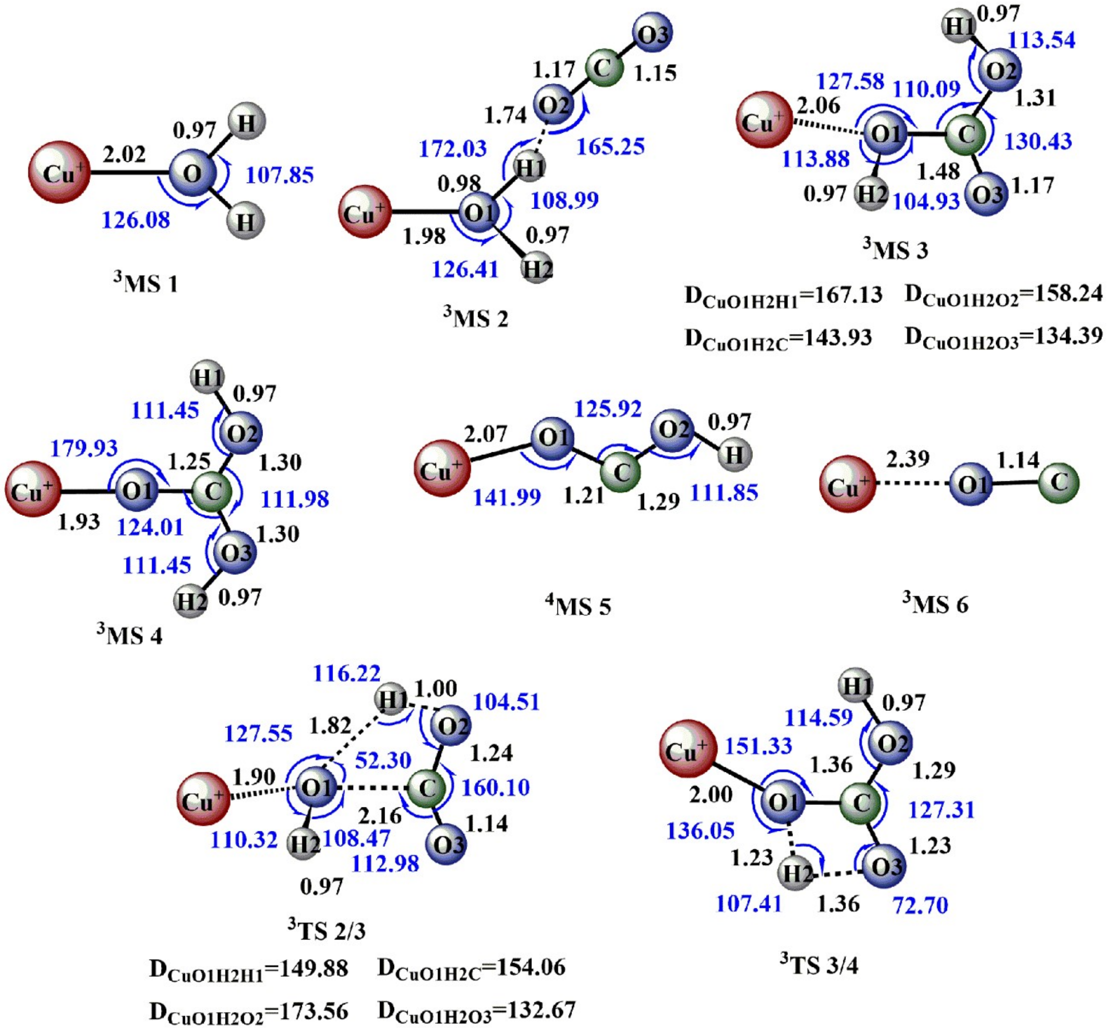

Supplementary Figure 34 | Optimal geometric structure of triplet intermediates and transition states of the reduction of CO_2 to CO catalyzed by on Cu^+ and H_2O at the B2PLYP/cc-PVTZ/Aug-cc-pVTZ-PP level. Bond lengths are in angstroms and angles in degrees.

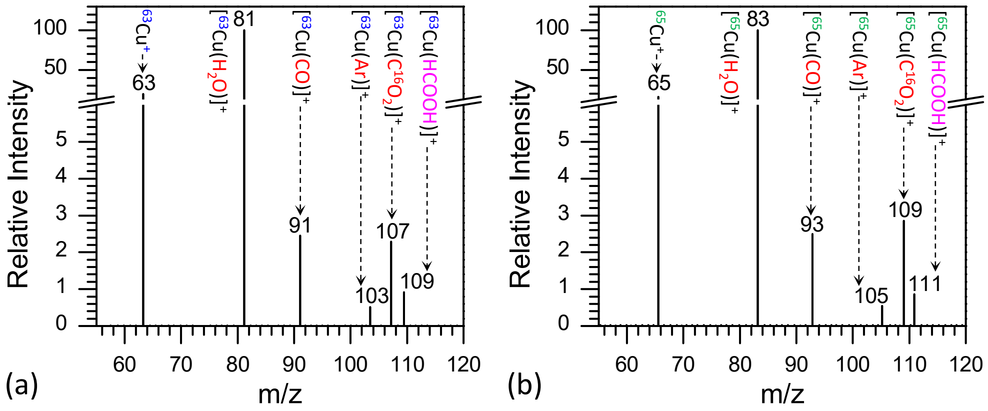

Supplementary Figure 35 | Mass spectra of CO_2 reduction to CO under different Cu-based catalytic systems. **(a)** $[^{63}Cu(H_2O)]^+$ and **(b)** $[^{65}Cu(H_2O)]^+$ (reaction temperature: 280 °C; reaction pressure: 1.5 mTorr).

Supplementary Tables 1-4:

Supplementary Table 1 | Comparison of the reaction conditions among TSQ mass spectrometer, realistic thermal (reverse water-gas shift reaction) and electrochemical catalysis.

Reaction System	Reaction Phase	Bulk Phase	Gas Component	Catalyst	Temperature	Pressure
TSQ system	gas phase	Ar gas	5% (v/v) CO_2/Ar	Cu, Ag or Pd-related ions	280 °C☆	1.3 x 10^{-7} MPa (1 mTorr)
realistic thermal catalysis	gas/solid phase	H_2 gas	9:1 (p/p) H_2/CO_2	Cu/ZnO/Al_2O_3	230 °C	3.0 MPa
electrochemical catalysis	gas/liquid phase	Ar gas/KCl solution	100% CO_2	Ag/Au/Pd electrodes	room temperature	0.1 MPa (1 atm)

Note: ☆ means the gas circuit temperature, rather than the reaction cell Q2 temperature, as shown in Fig. 1.

From **Supplementary Table 1**, it is apparent that the reaction conditions for CO_2RR using our modified TSQ mass spectrometer, realistic thermal (reverse water-gas shift reaction) and electrochemical catalysis have much difference. Despite this, the common point among them is that all the reactions between CO_2 and H_2O occur at the metal-based catalyst interfaces, which paves the way for studying the effect of H_2O on the efficiency of CO_2RR.

Supplementary Table 2 | Total (a.u.) and relative (kcal/mol) energies of the reaction species at B2PLYP/cc-pVTZ/Aug-cc-pVTZ-PP levels with inclusion of B2PLYP/cc-pVTZ/Aug-cc-pVTZ-PP zero-point vibrational energies (ZPVE).

Species	HF (a.u.)	ZPVE (kcal/mol)	E (a.u.)	G (a.u.)
$^{1}Cu^{+}$	-196.614697	0.00000	-196.770812	-196.786667
$^{3}Cu^{+}$	-196.557289	0.00000	-196.679126	-196.696018
^{1}CO	-113.165547	3.08798	-113.279127	-113.298255
$^{1}CO_2$	-188.342359	7.25625	-188.530740	-188.551442
$^{1}H_2O$	-76.323475	13.46352	-76.385495	-76.403133
^{2}H	-0.498614	0.00000	-0.498614	-0.509268

Supplementary Table 3 | Total (a.u.) and relative (kcal/mol) energies of the reaction species and transition states for CO_2 reduction catalyzed by singlet Cu^+ at B2PLYP/cc-PVTZ/Aug-cc-pVTZ-PP levels with inclusion of B2PLYP/cc-PVTZ/Aug-cc-pVTZ-PP zero-point vibrational energies (ZPVE).

Species	HF (a.u.)	ZPVE (kcal/mol)	E (a.u.)	ΔE (kcal/mol)	G (a.u.)	ΔG (kcal/mol)
^{1}IS 0	-272.938172	13.46352	-273.156307	0.00	-273.189800	0.00
^{1}MS 1	-273.002697	14.87082	-273.228003	-44.99	-273.253831	-40.18
^{1}MS 2	-461.356685	23.31149	-461.770379	-52.29	-461.805512	-40.33
^{1}TS 2-3	-461.267781	22.61025	-461.694034	-4.38	-461.725184	10.08
^{1}MS 3	-461.304744	24.84089	-461.714764	-17.39	-461.745771	-2.84
^{1}TS 3-4	-461.259195	22.12516	-461.679940	4.46	-461.711385	18.74
^{1}MS 4	-461.343945	25.63922	-461.749291	-39.06	-461.780343	-24.54
^{2}MS 5	-385.525099	14.19875	-385.864404	-40.31	-385.894068	-29.30
^{1}MS 6	-309.803547	3.53377	-310.077099	-102.79	-310.097232	-90.19
^{1}FS 7	-309.780244	3.08798	-310.049939	-85.75	-310.084922	-82.46

Supplementary Table 4 | Total (a.u.) and relative (kcal/mol) energies of the reaction species and transition states for CO_2 reduction catalyzed by triplet Cu^+ at B2PLYP/cc-PVTZ/Aug-cc-pVTZ-PP levels with inclusion of B2PLYP/cc-PVTZ/Aug-cc-pVTZ-PP zero-point vibrational energies (ZPVE).

Species	HF (a.u.)	ZPVE (kcal/mol)	E (a.u.)	ΔE (kcal/mol)	G (a.u.)	ΔG (kcal/mol)
^{3}IS-0	-272.880764	13.46352	-273.064621	57.53	-273.099151	56.88
^{3}MS-1	-272.936434	14.87574	-273.122875	20.98	-273.148695	25.79
^{3}MS-2	-461.291692	23.07575	-461.666555	12.86	-461.702649	24.22
^{3}TS2-3	-461.208354	22.47422	-461.593410	58.76	-461.625784	72.45
^{3}MS-3	-461.236631	24.42137	-461.608748	49.13	-461.641171	62.80
^{3}TS3-4	-461.192597	21.99768	-461.574184	70.82	-461.606112	84.80
^{3}MS-4	-461.280926	25.46224	-461.646256	25.60	-461.679847	38.53
^{4}MS-5	-385.454389	13.79788	-385.754578	28.61	-385.785273	38.97
^{3}MS-6	-309.733817	3.42056	-309.969051	-34.99	-309.996318	-26.86
^{3}FS-7	-309.722836	3.08798	-309.958253	-28.22	-309.994273	-25.58

Supplementary References

1 Sun, X., Wang, P., Shao, Z., Cao, X. & Hu, P. A first-principles microkinetic study on the hydrogenation of carbon dioxide over Cu(211) in the presence of water. *Sci. China Chem.* **62**, 1686-1697 (2019).

2 Bansode, A., Tidona, B., von Rohr, P. R. & Urakawa, A. Impact of K and Ba promoters on CO_2 hydrogenation over Cu/Al_2O_3 catalysts at high pressure. *Catal. Sci. Technol.* **3**, 767-778 (2013).

3 Rienstra-Kiracofe, J. C., Tschumper, G. S., Schaefer, H. F., Nandi, S. & Ellison, G. B. Atomic and molecular electron affinities: photoelectron experiments and theoretical computations. *Chem. Rev.* **102**, 231-282 (2002).

4 Boxford, W. E., Burke, R. M. & Dessent, C. E. H. New insights into dianion-cation contact ion-pairs: understanding the effect of cation complexation on the electron detachment and ionic fragmentation pathways of multiply charged anions. *Phys. Scr.* **76**, C56-C62 (2007).

5 Butcher, C. P. G. *et al.* Collision-induced dissociation and photodetachment of singly and doubly charged anionic polynuclear transition metal carbonyl clusters: $Ru_3Co(CO)_{13}^-$, $Ru_6C(CO)_{16}^{2-}$, and $Ru_6(CO)_{18}^{2-}$. *J. Chem. Phys.* **116**, 6560-6566 (2002).

6 Hunton, D. E., Albertoni, C. R., Märk, T. D. & Castleman, A. W. Unimolecular decay of metastable CO_3^-. *Chem. Phys. Lett.* **106**, 544-549 (1984).

7 Wu, R. L. C. & Tiernan, T. O. Evidence for excited states of CO_3^{-*} and NO_3^{-*} from collisional dissociation processes. *Planet. Space Sci.* **29**, 735-739 (1981).

8 Kim, J. Y., Rodriguez, J. A., Hanson, J. C., Frenkel, A. I. & Lee, P. L. Reduction of CuO and Cu_2O with H_2: H embedding and kinetic effects in the formation of suboxides. *J. Am. Chem. Soc.* **125**, 10684-10692 (2003).

9 Kim, D. *et al.* Insights into an autonomously formed oxygen-evacuated Cu_2O electrode for the selective production of C_2H_4 from CO_2. *Phys. Chem. Chem. Phys.* **17**, 824-830 (2015).
